# Premature morbidity and mortality associated with potentially undiagnosed familial hypercholesterolemia in the general population

**DOI:** 10.1016/j.ajpc.2023.100580

**Published:** 2023-09-06

**Authors:** Kausik K. Ray, Demetris Pillas, Savvas Hadjiphilippou, Kamlesh Khunti, Sreenivasa Rao Kondapally Seshasai, Antonio J. Vallejo-Vaz, David Neasham, Janet Addison

**Affiliations:** a Imperial Centre for Cardiovascular Disease Prevention (ICCP), Dept. of Primary Care and Public Health, School of Public Health, Imperial College London, Charing Cross Campus, The Reynolds Building, St Dunstan's Road, London W6 8RP, United Kingdom; b Amgen Ltd, Uxbridge, United Kingdom; c University of Leicester, Leicester, United Kingdom; d Cardiovascular Clinical Academic Group, Molecular and Clinical Sciences Research Institute, St George's, University of London and St George's University Hospitals NHS Foundation Trust, London, United Kingdom; e Department of Medicine, Faculty of Medicine, University of Seville, Seville, Spain; f Clinical Epidemiology and Vascular Risk, Instituto de Biomedicina de Sevilla, IBiS/Hospital Universitario Virgen del Rocío/Universidad de Sevilla/CSIC. Seville, Spain; g Biogen Idec Ltd, Maidenhead, United Kingdom

**Keywords:** Familial hypercholesterolemia, Cardiovascular disease prevention, LDL-C, Screening

## Abstract

**Background:**

Familial hypercholesterolemia (FH) is common, but underdiagnosed, and few systematic early screening programs exist.

**Objective:**

To assess health outcomes among those with a recorded diagnosis of FH and potential cases of FH with no recorded diagnosis.

**Methods:**

Retrospective cohort study using the UK Clinical Practice Research Datalink. Records of adults were classified as diagnosed FH (FH_Coded_), or via accepted algorithms using LDL-C and clinical characteristics as potential FH (FH_Potential_) or unlikely FH (FH_Unlikely_) using the DLCN or EUROASPIRE criteria (but no record of FH). Outcomes assessed were premature cardiovascular (CV) events, premature deaths and life expectancy.

**Results:**

Among 1,729,046 individuals free from CV events, a record of FH_Coded_ before the age of 40 was 0.3/1000 (IQR 0.3–0.4) and increased with age. Where LDL-C levels were available, 1.8/1000 (IQR 1.6–2.0) could be classified as FH_Potential_. LDL-C was higher for both FH_Coded_ and FH_Potential_ vs FH_Unlikely_ (185.6 and 216.6 vs 116 mg/dL, respectively, *p*<0.001). Compared to FH_Unlikely_ both FH_Coded_ and FH_Potential_ cohorts had a higher risk of premature cardiovascular events (both *p*<0.001) with highest rates among FH_Coded_. Risk of premature deaths did not differ between FH_Coded_ and FH_Unlikely,_ but was 1.88 (95% CI 1.27–2.78, *p* = 0.002) for FH_Potential_ vs FH_Coded_ and 2.40 (95% CI 1.57–3.67, *p*<0.001) for FH_Potential_ vs FH_Unlikely_. At age 18, the FH_Potential_ cohort had a life expectancy 16 years lower than the FH_Coded_ cohort (*p*<0.001).

**Conclusions:**

Potential cases of FH had a doubling in risk of premature death and a large reduction in life expectancy compared to individuals with a recorded diagnosis of FH. These findings strengthen the critical importance of identifying potential cases of FH early and early treatment.

## 1 Introduction

FH is an autosomal co-dominant genetic disease mostly due to mutations in the LDL-R, ApoB or PCSK9 genes*.* It has a prevalence of 1:311 in general populations without founder effects [1] and is associated with a cumulative exposure to elevated low density lipoprotein cholesterol (LDL-C) levels from birth, resulting in an increased risk of premature cardiovascular disease [2]. Despite these data [3], FH remains under-diagnosed thus representing a major public health challenge [2]. As the cardiovascular consequences of FH are largely silent until its major clinical manifestation with premature cardiovascular mortality and morbidity [4,5], altering its natural history relies upon early detection and initiation of lipid-lowering therapies and behavioral/lifestyle changes [2,6]. That said, there is currently considerable debate about the merits of screening and how best to achieve it, with some advocating universal screening while others proposing cascade screening of index cases [7], [8], [9], [10], [11]. Beyond currently available data, the case for screening for FH could be further supported if the natural history of undiagnosed cases of FH (and, by inference, delayed diagnosis/treatment), were shown to adversely impact mortality and morbidity. Hence, we compared health outcomes between adults undiagnosed but potentially having FH, versus those with a recorded diagnosis of FH and those unlikely to have FH. We hypothesized that potential cases of FH in whom there was no recorded diagnosis would have worse health outcomes compared to those in whom a diagnosis of FH was unlikely or those where a diagnosis of FH was present.

### 1.1 Subjects and methods

#### 1.1.1 Study design and data sources

This retrospective cohort study utilized data from UK CPRD - a nationwide, longitudinal, electronic database initiated in 1987 and shown to be representative of the UK population [12]. CPRD houses data for over 10 million individuals, using Read codes as standard terminology, and, where linkages allow, supplemented with additional data from Hospital Episodes Statistics (HES; Supplemental Methods) and mortality data from the Office of National Statistics (ONS; Supplemental Methods). Scientific approval was granted by the CPRD Independent Scientific Advisory Committee.

### 1.2 Study patients

Adults (≥ 18 years of age) registered in CPRD within the 5-year period from August 1, 2008 (the date of introduction of National Institute for Health and Care Excellence (NICE) guidance on FH diagnosis) [13] to July 31, 2013 were eligible for inclusion. Utilizing all available CPRD data (from date of first CPRD record to July 31, 2013), three cohorts were defined. Supplemental Methods Tables 1 and 2 provide details of variables that were available and allowed for the cohorts to be created. The three cohorts were: (i) FH_Coded_: individuals with FH diagnosis identified by READ codes (Supplemental Methods Table 1) [14]; (ii) FH_Potential_: individuals with at least one LDL-C value and no Read-coded FH diagnosis, but who could potentially be considered to have FH based on achieving a ‘Definite’ or ‘Probable’ score calculated according to either the Dutch Lipid Clinic Network (DLCN) (Supplemental Methods Table 2) [2] or EUROASPIRE modified DLCN criteria which accounts for lipid lowering therapy at baseline (Supplemental Methods Table 3) [15], or both; hence individuals who are not on lipid lowering therapy would be captured via the DLCN criteria and those on lipid lowering treatment by EUROASPIRE (iii) FH_Unlikely_: individuals with at least one LDL-C record and not meeting criteria (i) or (ii). Thus, every person in FH_Potential_ or FH_Unlikley_ category had at least one LDL-C measurement. If a variable contributing to DLCN or EUROASPIRE data was not recorded in CPRD, then we assumed the characteristic to be absent. Potential secondary causes were not specifically excluded. No READ codes specific for homozygous FH were included.

We excluded individuals with a prior history of cardiovascular disease at baseline in each of the three cohorts (FH_Coded_, FH_Potential_, and FH_Unlikely_) to mitigate reverse association bias. Hence, to be defined as FH_Potential_ an individual would require a higher LDL-C level to cross the threshold of being defined as FH_Potential_. Without this restriction, an individual might cross the threshold for FH_Potential_ with a lower LDL-C level and a positive history of premature cardiovascular disease. Using this approach, we used the FH_Coded_ and FH_Potential_ cohorts to act as proxies for diagnosed and undiagnosed FH, respectively.

For the FH_Coded_ cohort, study baseline was defined as the date of the first diagnostic FH Read code record in CPRD. For FH_Potential_, baseline was set as the date at which a patient attained the threshold classification score according to the algorithm. For FH_Unlikely_, baseline was set at August 1, 2008. Baseline demographics were extracted from CPRD and HES.

### 1.3 Outcomes

Outcomes of interest included age-specific cardiovascular event rate, premature cardiovascular events, life expectancy, premature mortality and premature cardiovascular mortality occurring during the individual follow-up period from patient entry into CPRD to July 31, 2014. A cardiovascular event was defined as any of the following atherosclerosis-related outcomes (fatal or non-fatal): unstable angina, acute myocardial infarction, ischemic stroke, transient ischemic attack, coronary revascularization, and peripheral arterial disease, identified by CPRD Read Codes (Supplemental Methods Table 4) and HES ICD-10 codes (HES; Supplemental Methods). Premature cardiovascular events were defined as those occurring prior to 55 and 60 years of age in males and females, respectively, and premature death occurring below the age of 60 in either gender [2]. Cause-specific mortality and date of death were identified in ONS, or in CPRD for patients not linked to ONS.

### 1.4 Statistical analysis

The proportion of patients in CPRD meeting the definitions of FH_Coded_ and FH_Potential_ cohorts was estimated. Age-specific cardiovascular event rates were calculated for the three cohorts after excluding individuals with prior cardiovascular disease at baseline, as described above. Differences in incidence and risk of premature cardiovascular events were estimated through Incidence Rate Ratios (IRRs) and Hazard Ratios (HRs). The IRRs of premature cardiovascular events were calculated for the FH_Coded_ and FH_Potential_ cohorts vs FH_Unlikely_ and for FH_Potential_ vs FH_Coded_. HRs of experiencing a cardiovascular or a premature cardiovascular event were estimated for FH_Coded_ and FH_Potential_ vs FH_Unlikely_, for all patients and by age and sex. HRs were estimated using the PHREG command in SAS v.9.4, that fits a superset of the Cox model, known as the multiplicative hazards model. Student t-tests were performed to evaluate between-group differences for continuous variables.

Mortality rates for all deaths were age- and sex-standardized to the European Standard Population, and life-expectancy estimates were calculated using the Chiang II method (Supplemental Methods) [16]. The contribution of circulatory-related deaths (ICD-10 Chapter 9) to the mortality rates of the FH_Coded_ and FH_Potential_ cohorts was estimated as a percentage of the mortality rate difference between the two groups. The proportion of the risk of premature death in the FH_Coded_ and FH_Potential_ groups attributable to being classified as FH_Potential_ was calculated (Population Attributable Risk Percentage (PAR%); Supplemental Methods). A complete list of causes of death included under “mortality attributable to circulatory causes” are listed in the supplemental data (Supplemental Methods Table 6) of which the vast majority were related to atherosclerotic cardiovascular disease.

To evaluate the extent to which differences in outcomes might be due to imbalances in baseline risk factors, sensitivity analyses excluding patients with a history of diabetes and/or hypertension, and current smokers were conducted. No imputation for missing data was performed. Analyses were conducted using SAS software, version 9.4 (SAS Institute).

## 2 Results

### 2.1 Study population

There were 1,729,046 individuals without cardiovascular disease at baseline who fulfilled our inclusion criteria (Supplemental Figure 1). The proportion of individuals in the FH_Coded_ and FH_Potential_ cohorts were 1.3 (95% CI: 1.2–1.3) and 7.8 (95% CI: 7.7–7.9) per thousand, and were lowest below the age of 40 (*p*<0.001) (Supplemental Table 1). Coding for FH was lowest in Northern Ireland, 0.6 per 1000 (95% CI 0.5–0.8), and highest in Wales at 3.0 per 1000 (95% CI 2.8–3.1). Approximately 45% of the FH_Coded_ and 42% of the FH_Potential_ cohorts were male; the FH_Coded_ cohort were on average 10 years younger and less likely to be smokers, have diabetes or hypertension (*p*<0.001 for all) vs other cohorts (Table 1).

**Table 1:** Baseline Characteristics of Patients Stratified by FH Cohort.

	FH_Coded_	FH_Potential_	FH_Unlikely_
	*n* = 6843 (0.4%)	*n* = 13,459 (0.8%)	*n* = 1708,744 (98.8%)
Demographic Characteristics			
Age, years, mean (SD)	50.9 (13.7)	60.8 (11.9)	62.2 (15.8)
Gender, n (%)			
Male	3090 (45.2)	5641 (41.9)	818,190 (47.9)
Female	3753 (54.8)	7818 (58.1)	890,549 (52.1)
Ethnicity, n (%)			
White	3550 (51.9)	4284 (31.8)	540,432 (31.6)
Region, n (%)			
England	4947 (72.3)	10,250 (76.2)	1,393,334 (81.5)
Scotland	480 (7.0)	902 (6.7)	85,854 (5.0)
Wales	1325 (19.4)	1587 (11.8)	166,681 (9.8)
Northern Ireland	91 (1.3)	720 (5.3)	62,875 (3.7)
Clinical Characteristics			
BMI, n (%)			
Normal weight (BMI: <25)	1045 (15.3)	2391 (17.8)	391,508 (22.9)
Overweight (BMI: 25–29.9)	1479 (21.6)	4532 (33.7)	480,077 (28.1)
Obese (BMI: 30+)	964 (14.1)	4031 (30.0)	431,895 (25.3)
Smoking, n (%)			
Never smoked	2185 (31.9)	5781 (43.0)	786,728 (46.0)
Previous smoker	983 (14.4)	3547 (26.4)	456,120 (26.7)
Current smoker	914 (13.4)	3001 (22.3)	239,269 (14.0)
Diabetes, n (%)	120 (1.8)	1890 (14.0)	157,237 (9.2)
Hypertension, n (%)	829 (12.1)	4064 (30.2)	299,518 (17.5)
Family History	567 (8.3)	1226 (9.1)	54,680 (3.2)
Lipids (mg/dL) LDL cholesterol level	185.6 (58)	216.6 (50.3)	116 (38.7)
HDL cholesterol	58 (65.7)	61.9 (123.7)	58 (61.9)
Triglycerides	186 (IQR115.2–310)	212.6 (IQR 141.7–487.2)	115.2 (IQR) 97.4–132.9)
Statin prescription, n (%)	2280 (33.3)	9084 (67.5)	413,776 (23.9)
High- intensity statin prescription, n (%)	771 (11.3)	2756 (20.5)	48,428 (2.8)

Lipids are shown as mean and standard deviation (SD), or median and interquartile range (IQR). The total number for each baseline characteristic reflects recording of variables in the CPRD database. BMI= body-mass index, ESC= European Society of Cardiology, FH= familial hypercholesterolemia, HDL= high density lipoprotein, LDL= low-density lipoprotein.

Of the 1708,744 in the FH_Unlikely_ cohort 5.6% (95,689) could be classified as possible FH, thus the majority could be considered as true FH_Unlikely._ The FH_Potential_ cohorts were derived from 2873 individuals who fulfilled the DLNC for probable or definite FH and 10 586 individuals using the equivalent EUROASPIRE criteria. Of those considered as FH_Potential_ through the DLCN criteria, the major drivers were high LDL-C (71.8%) followed by family history (26.2%) (Supplemental Table 2). The corresponding figures for those conforming to the EUROASPIRE criteria were high LDL-C in 95.5% and family history in 4.5%. (Supplemental Table 2). The proportion of individuals who could be considered definite by DLCN criteria were 6% and by EUROASPIRE criteria 7.7% respectively, thus the majority of individuals in the FH_Potential_ category would be considered as consisting of probable FH by either criteria (Supplemental Table 3).

### 2.2 Lipids

**Fig. 1:**
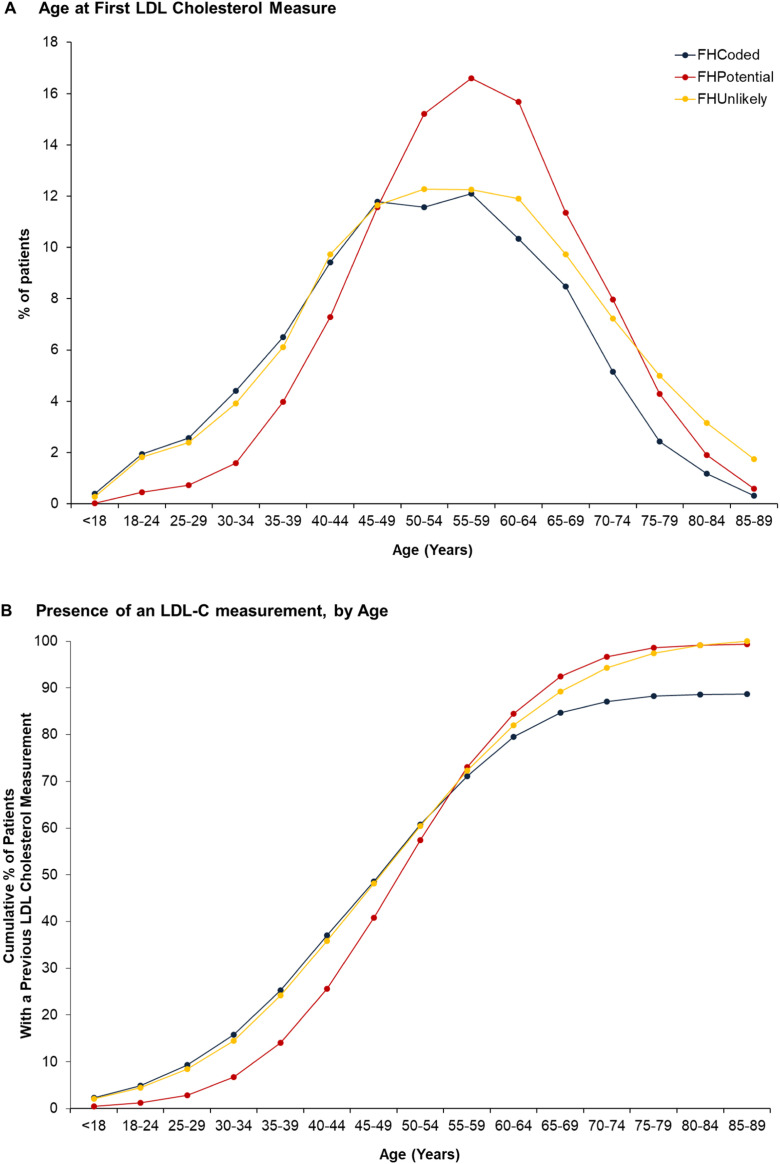
The Timing of First Cholesterol Measurements by Age Across Cohorts. Panel A: Age at first LDL Cholesterol Measurement. Panel B: LDL Cholesterol Measurement by age. On average, the FH_Potential_ cohort were 4.3 years (95% CI: 4.0–4.7, *p*<0.001) older than the FH_Coded_ cohort at time of first recorded LDL-C measurement. Among the FH_Coded_ cohort, 25% had a LDL-C record by the age of 45 and 50% by age 55. FH= Familial Hypercholesterolemia.

On average, the FH_Potential_ cohort were 4.3 years (95% CI: 4.0–4.7, *p*<0.001) older than the FH_Coded_ cohort at time of first recorded LDL-C measurement (Fig. 1A). Among the FH_Coded_ cohort, 25% had a LDL-C record by the age of 45 and 50% by age 55 (Fig. 1B). Among the FH_Potential_ cohort the corresponding figures were 50 and 58 years, respectively. At baseline, recorded LDL-C levels were 185.6 ± 58 mg/dL and 216.6 ± 50.3 mg/dL for FH_Coded_ and FH_Potential_, respectively (Table 1.) with FH_Unlikely_ having lower levels (116 ± 38.7 mg/dL) (*p*<0.001). Recorded statin use at baseline varied between groups with 33.3% of FH_Coded_ recording statin use (of which 11.3% was high intensity) versus 67.5% (of which 20.5% was high intensity) among the FH_Potential_.

### 2.3 FH and cardiovascular events

**Fig. 2:**
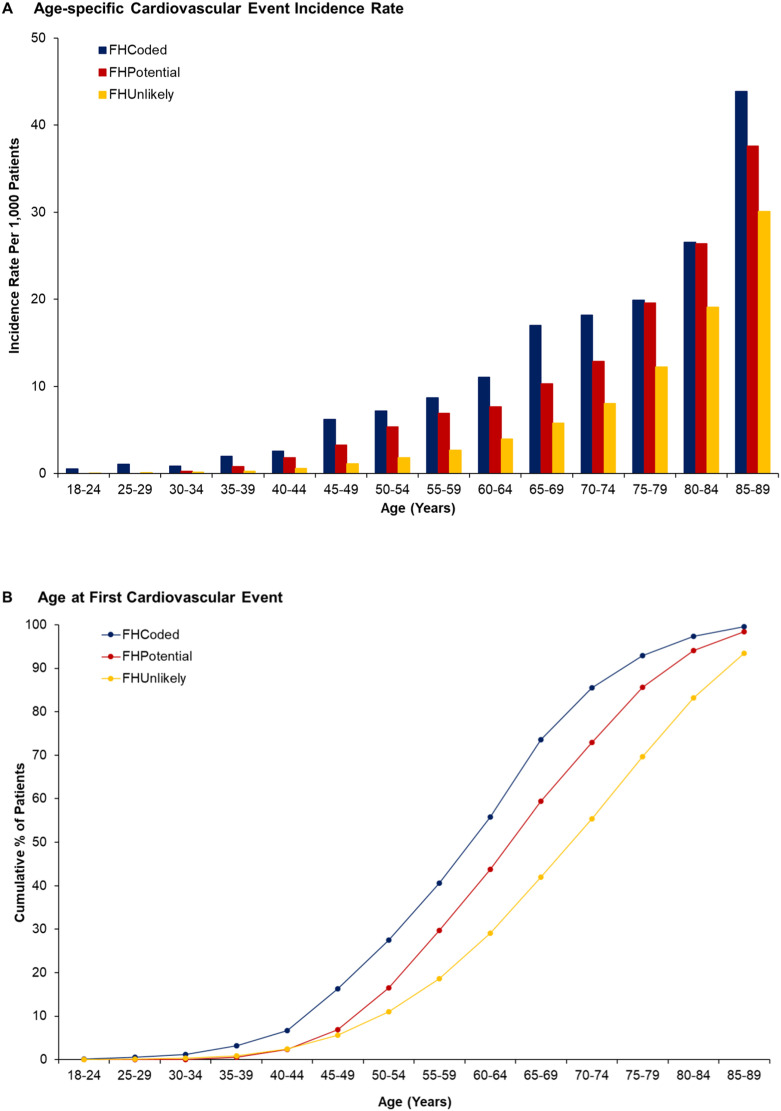
The Occurrence of Cardiovascular Events by Age Across Cohorts. Panel A: Age-specific Cardiovascular Event Incidence Rate. Panel B: Age at First Cardiovascular Event. The age specific incidence of fatal and non-fatal cardiovascular events increased with age for all three cohorts and was highest for FH_Coded_ and lowest for FH_Unlikely_ within each age category. Of first cardiovascular events, 40.6% occurred by the age of 60 in the FH_Coded_ cohort compared with 29.7% and 18.6% in the FH_Potential_ and FH_Unlikely_ cohorts, respectively (*p*<0.001). FH= Familial Hypercholesterolemia.

The incidence of cardiovascular events increased with age for all three cohorts and was highest for FH_Coded_ and lowest for FH_Unlikely_ within each age category (Fig. 2A and Supplemental Table 4). Compared to FH_Unlikely_, the risk of cardiovascular events was significantly higher for both FH_Coded_ and FH_Potential_ from age 40 onwards (Table 2). Compared with FH_Unlikely_, the risk of premature cardiovascular events was significantly higher for both FH_Coded_ and FH_Potential_ (HR 4.21, 95% CI 3.69–4.81 and HR 3.46, 95% CI 3.07–3.89 respectively). Of first cardiovascular events, 40.6% occurred by the age of 60 in the FH_Coded_ cohort compared with 29.7% and 18.6% in the FH_Potential_ and FH_Unlikely_ cohorts, respectively (*p*<0.001) (Fig. 2B). In sensitivity analyses excluding individuals with diabetes, hypertension and current smokers, the risk of premature cardiovascular events was lower among FH_Potential_ 0.62 (95% CI 0.45–0.86) than for FH_Coded_. However, the risk of premature cardiovascular events among the FH_Potential_ cohort were higher than for FH_Unlikely_ (RR 1.95, 95% CI 1.53–2.48) after excluding individuals with the same risk factors (Supplemental Table 5). Compared to FH_Unlikely_ premature cardiovascular events increased in a graded fashion among the FH_Potential_ cohort with probable FH having a HR of 3.08 (95% CI 2.70–3.52) and definite FH having a HR of 6.50 (95% CI 5.03–8.39), *p* <0.001 for both (Supplemental Table 6).

**Table 2:** Hazard Ratios of Cardiovascular Events by Age for Coded and Potential FH vs Unlikely, and Overall Cardiovascular events and Premature Cardiovascular Event stratified by Gender.

	Hazard Ratio		Hazard Ratio	
	(FH_Coded_ vs FH_Unlikely_)		(FH_Potential_ vs FH_Unlikely_)	
Age (years)	HR (95% CIs)	P-value	HR (95% CIs)	P-value
18–24	N/A	N/A	N/A	N/A
25–29	N/A	N/A	N/A	N/A
30–34	N/A	N/A	N/A	N/A
35–39	0.80 (0.11, 5.73)	0.827	1.87 (0.26, 13.35)	0.531
40–44	2.99 (1.60, 5.59)	<0.001	3.29 (1.56, 6.92)	0.002
45–49	3.02 (2.02, 4.52)	<0.001	3.27 (2.19, 4.90)	<0.001
50–54	2.64 (1.94, 3.61)	<0.001	3.78 (3.00, 4.77)	<0.001
55–59	3.01 (2.35, 3.86)	<0.001	2.91 (2.45, 3.46)	<0.001
60–64	2.53 (2.02, 3.17)	<0.001	2.11 (1.79, 2.49)	<0.001
65–69	1.97 (1.60, 2.43)	<0.001	1.80 (1.56, 2.08)	<0.001
70–74	2.12 (1.72, 2.60)	<0.001	1.69 (1.47, 1.94)	<0.001
75–79	2.17 (1.78, 2.64)	<0.001	1.57 (1.36, 1.81)	<0.001
80–84	2.31 (1.90, 2.81)	<0.001	1.43 (1.23, 1.66)	<0.001
85–89	1.90 (1.46, 2.46)	<0.001	1.34 (1.11, 1.62)	0.002
All Cardiovascular events				
All patients	1.97 (1.82, 2.12)	<0.001	1.89 (1.79, 1.99)	<0.001
Male	2.04 (1.84, 2.26)	<0.001	1.84 (1.70, 1.99)	<0.001
Female	1.95 (1.74, 2.17)	<0.001	2.04 (1.89, 2.19)	<0.001
Premature Cardiovascular event				
All patients	4.21 (3.69, 4.81)	<0.001	3.46 (3.07, 3.89)	<0.001
Male	4.35 (3.66, 5.18)	<0.001	3.37 (2.86, 3.96)	<0.001
Female	3.94 (3.20, 4.85)	<0.001	3.61 (3.04, 4.28)	<0.001

CI= confidence interval, CV= cardiovascular, FH= familial hypercholesterolemia, HR= hazard ratio.

### 2.4 FH and premature mortality

The proportion of premature deaths attributable to circulatory causes was similar for FH_Coded_ (30.0%) and FH_Potential_ (31.9%) and higher than in the FH_Unlikely_ cohort (9.5%) (Table 3). Standardized mortality rates were highest among FH_Potential_ and lowest among FH_Unlikely_, but were not significantly different for FH_Coded_ vs FH_Unlikely_ (*p* = 0.689). In contrast, the risk of premature deaths among the FH_Potential_ was seven-fold higher vs FH_Unlikely_ (RR 7.30, 95% CI 5.26–10.11, *p*<0.001) and sixfold higher vs FH_Coded_ (RR 6.69, 95% CI 4.89–9.15, *p*<0.001). Although the elevated risk of premature deaths among FH_Potential_ vs FH_Coded_ was in part related to comorbidities and attenuated considerably in sensitivity analyses which excluded individuals with diabetes, hypertension and smoking at baseline, it remained significant (RR 1.88, 95% CI 1.27–2.78, *p* = 0.002) (Table 3). Corresponding figures for FH_Potential_ vs FH_Unlikely_ were RR 2.40, 95% CI 1.57–3.67, *p*<0.001. Compared to FH_Coded_ premature deaths increased in a graded fashion among the FH_Potential_ cohort with probable FH having a HR of 2.99 (95% CI 1.73–5.16) and definite FH having a HR of 3.71 (95% CI 1.68–8.18), *p* <0.001 for both (Supplemental Table 7).The population attributable risk of premature death among the combined cohort with elevated LDL-C (FH_Coded_ and FH_Potential_) attributed to the FH_Potential_ cohort was 67.53% overall and 55.58% after excluding people with diabetes, hypertension and current smoking (Supplemental Table 8).

**Table 3:** Standardized Mortality Rates and Relative Risks of Premature Deaths (<60 years).

	Original Result	Sensitivity Analysis I	Sensitivity Analysis II
		Excluding Diabetes and Hypertension	Excluding Diabetes, Hypertension, and Smoking
	Standardized Mortality Rate per 100,000 (95% Cis)	Standardized Mortality Rate per 100,000 (95% Cis)	Standardized Mortality Rate per 100,000 (95% Cis)
Premature Mortality			
FH_Coded_	44.6 (26.4, 62.8)	40.1 (16.4, 63.7)	38.3 (13.3, 63.4)
FH_Potential_	298.4 (240.8, 356.0)	125.4 (79.8, 171.1)	72.6 (34.6, 110.6)
FH_Unlikely_	40.9 (40.0, 41.8)	38.2 (37.1, 39.4)	30.2 (29.1, 31.3)
Relative Risk (FH_Potential_ vs FH_Coded_)	6.69 (4.88, 9.16), *p*<0.001	3.13 (2.19, 4.46), *p*<0.001	1.88 (1.27, 2.78), *p* = 0.002
Relative Risk (FH_Potential_ vs FH_Unlikely_)	7.30 (5.26, 10.11), *p*<0.001	3.28 (2.29, 4.72), *p*<0.001	2.40 (1.57, 3.67), *p*<0.001
Premature Mortality attributable to circulatory causes		Proportion Attributable to Circulatory Causes	
FH_Coded_	13.4 (3.5, 23.4)	30.0%	
FH_Potential_	95.1 (58.5, 131.6)	31.9%	
FH_Unlikely_	3.9 (3.7, 4.0)	9.5%	

Rates are per 100,000 population, and are age- and gender-standardized to the European Standard Population. Sensitivity analysis excluded patients with a history of diabetes and/or hypertension, and then current smoking. FH= familial hypercholesterolemia, RR= relative risk.

### 2.5 FH and life expectancy

**Fig. 3:**
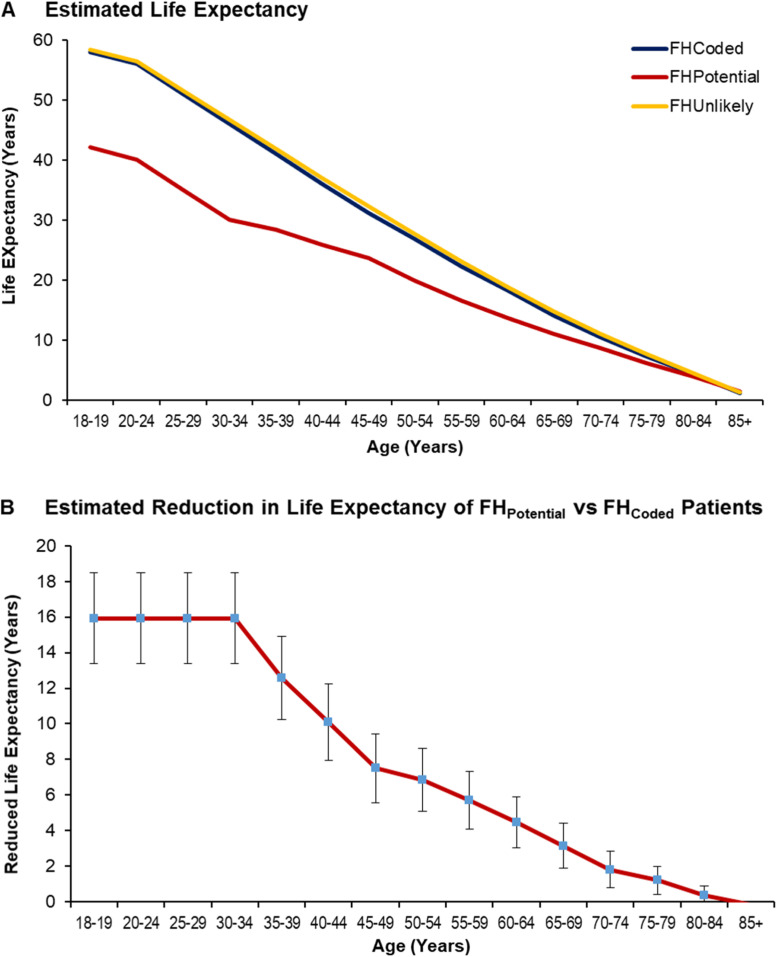
Life Expectancy at a Given Age by Cohort. Panel A: Estimated Life Expectancy. Panel B: Estimated Reduction in Life Expectancy. Compared with the FH_Coded_ cohort, life expectancy of individuals in the FH_Potential_ cohort was one and a half decades lower (15.9 years, 95% CI:13.4–18.5 years) for those aged 18–19 years, one decade lower (10.1 years, 95% CI:7.9–12.2) for those aged 40–44 years, and approximately half a decade lower (4.5 years, 95% CI:3.0–5.9) for those aged 60–64 years. There were no significant differences in life expectancy between the FH_Coded_ and FH_Unlikely_ cohorts. FH= Familial Hypercholesterolemia.

Compared with the FH_Coded_ cohort, life expectancy of individuals in the FH_Potential_ cohort was one and a half decades lower (15.9 years, 95% CI:13.4–18.5 years) for those aged 18–19 years, one decade lower (10.1 years, 95% CI:7.9–12.2) for those aged 40–44 years, and approximately half a decade lower (4.5 years, 95% CI:3.0–5.9) for those aged 60–64 years (Fig. 3A and B). There were no significant differences in life expectancy between the FH_Coded_ and FH_Unlikely_ cohorts (Supplemental Figure 2).

## 3 Discussion

A key premise to making a diagnosis of FH is that an LDL-C measurement is required as a minimum. This is supported by the observation the FH_Coded_ group were more likely to have their first LDL-C recorded before the age of 40 compared to the other two cohorts. That said, the proportion of individuals coded as FH before the age of 40 was low and increased with age, possibly reflecting a greater propensity for opportunistic diagnoses during interaction with the healthcare system or arising through cascade screening. Furthermore, there was considerable variation in recorded coding of FH across the United Kingdom, with Wales, which has an established screening program recording the highest coding for FH, and Northern Ireland, which lacks a formal strategy, recording the lowest. Taken together, these observations reinforce the role of public health policies on FH detection rates as well as the fact that most members of the general population are unlikely to have their first measurement of LDL-C before the age of 40.

In this study, mean baseline LDL-C values in the FH_Coded_ and FH_Potential_ cohorts were considerably higher than the FH_Unlikely_ cohort. Age-specific rates of fatal and non-fatal cardiovascular events increased cumulatively in all three studied cohorts but were generally highest at a given age among the FH_Coded_ then FH_Potential_ cohorts versus FH_Unlikely._ This is despite the older age and overall higher risk profile of the FH_Potential_ group suggesting that the FH_Coded_ were correctly identified as having the highest prevalence of patients with genetic variants causing FH and is consistent with higher, long-term LDL-C exposure [17]. Moreover the FH_Coded_ group appeared to be somewhat “undertreated” at baseline and had very high event rates reinforcing the need to both diagnosis and better treatment. The contribution of circulatory deaths to overall premature all-cause mortality was similar between FH_Potential_ and FH_Coded_ at around one third of all deaths; in contrast, circulatory deaths contributed to <10% of premature deaths in the FH_Unlikely_ cohort, consistent with exposure to lower life-long levels of LDL-C. Though the FH_Potential_ and FH_Coded_ cohorts were more similar when compared to the FH_Unlikely_ cohort, they differed in that age standardized risk of premature deaths were almost two fold higher among the FH_Potential_ cohort versus FH_Coded_ with differences in survival between FH_Potential_ and FH_Coded_ already apparent by the age of 18. Although the FH_Potential_ group were more likely to be older and have additional cardiovascular risk factors, these risk factors did not appear to explain the excess age standardized premature mortality.

The paradoxical findings of less statin use, less intensive statin prescription among FH_Coded_ Vs FH_Potential_ merit discussion. The most likely reason is that it is a function of the study design and the limitation of capturing changes in care that occurred after what was considered the baseline observation period. The design of this analysis required either a coded diagnosis of FH to create the FH_Coded_ cohort or an LDL-C measurement enabling us to create the FH_unlikely_ or FH_Potential_ groups. This means that the FH_Potential_ patients were older, more likely to have higher LDL-C levels as often the physical signs contributing to the DLCN criteria were not recorded. As they were older they were also more likely to have comorbidities and as a result may have been more likely to receive statins and possibly at a higher dose (bias by indication). Moreover, the baseline observation period may not account for treatment initiation or intensification, as part of shared care with specialist hospital clinics for those with a recorded diagnosis. In the UK, most patients interact with their GP and treatment may not have been initiated by the GP who may have suspected FH before referring to secondary care. If the diagnosis of FH were to be confirmed in secondary care then upon return to the GP, medication would have been initiated post the baseline observation period.

Reliable evidence supports the notion that LDL-C exposure is not only a causal risk factor for CVD but its effects are cumulative [18]. As LDL-C cumulative exposure tracks with atherosclerosis burden [19,20] and events [21], it follows that among those in whom treatment is initiated later there is already likely to be a greater underlying burden of atherosclerosis. Therefore, even if treatment is initiated, but occurs later, it may not be enough to mitigate part of the excess risk from the “missed cholesterol years [22].” Moreover, patients with low adherence to high-intensity statins have less LDL-C reduction and higher risk than patients with optimal adherence to low intensity statins [23], reinforcing the potential impact of behavior on outcomes beyond prescriptions alone. Those diagnosed as having FH (FH_Coded_) had LDL-C measurements earlier and were thus more likely to have treatment initiated earlier and potentially the first cardiovascular events which occurred, though more frequent, could have been less severe. In contrast, those who met criteria to be considered as potentially having FH, tended to be older and more likely had treatment initiated when older and thus have the potential for a greater burden of underlying atherosclerosis. It follows therefore that though apparently treated more intensively, the “missed cholesterol years” of exposure could contribute to worse survival if the first event that occurs were more likely to be fatal than a non-fatal event. This is consistent with our observation that among the FH_Potential_ group, a graded relationship was observed after separating this group into Probable FH and Definite FH.

Despite the significantly higher rates of premature fatal and non-fatal cardiovascular events and higher LDL-C levels, premature mortality rates were not statistically higher in the FH_Coded_ (diagnosed) cohort compared with FH_Unlikely_. The underlying reasons for this are unclear and could in part reflect survival bias with the sickest individuals having died prior to being eligible for our study as well as those with pre-existing cardiovascular disease being excluded (by study design). There was greater use of statins at baseline and fewer cardiovascular risk factors for the FH_Coded_ cohort compared with FH_Unlikely_. Even though these individuals had higher LDL-C, receiving a diagnosis of FH could result in favorable behavioral and lifestyle changes [24] which may contribute towards attenuating some of the risks related to FH, particularly if the diagnosis is made early and treatment initiated early as suggested by recent studies [22].

**Fig. 4:**
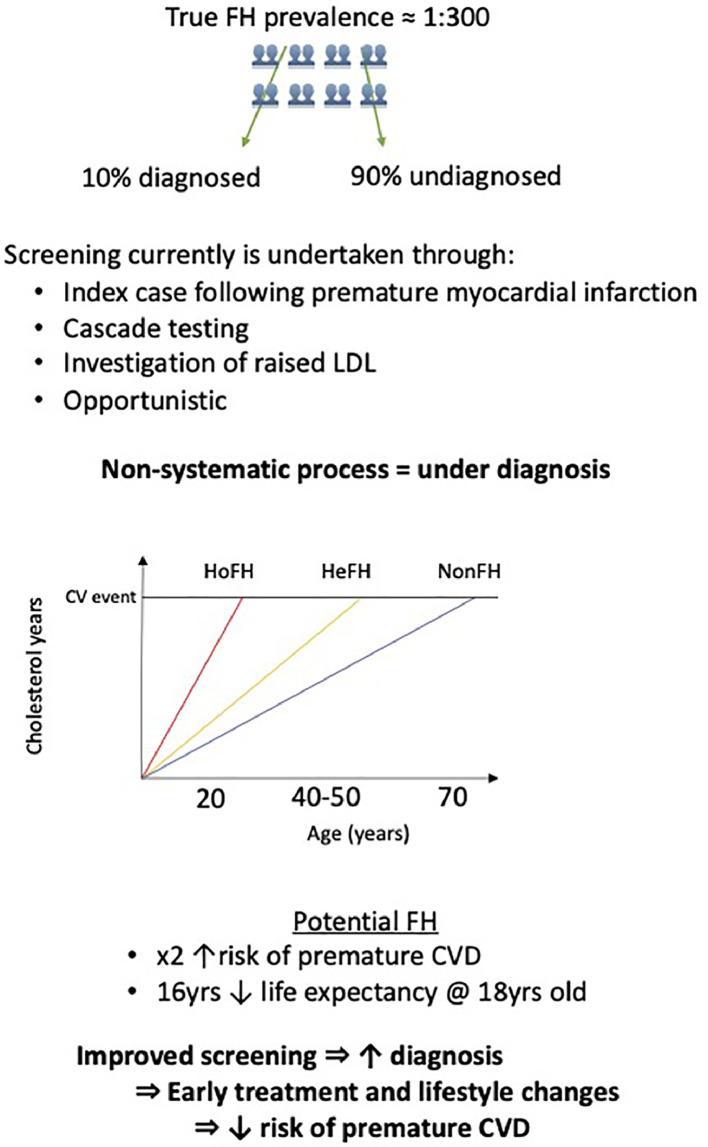
Central Illustration: Familial Hypercholesterolemia screening and diagnosis. Although familial hypercholesterolemia (FH) occurs in ∼1:300 individuals, few systematic screening programs exist. As a result, most cases of FH remain undiagnosed resulting in significant missed years of LDL exposure, and atherosclerotic burden. This increases the likelihood of subsequent premature cardiovascular disease and related morbidity and mortality. Patients with potentially undiagnosed FH have a two-fold increased risk of premature deaths with 16 years reduced life expectancy at age 18 compared to those with a confirmed diagnosis of FH. Early diagnosis and treatment is key to reducing the cardiovascular consequences of FH. CVD= cardiovascular disease, FH= Familial Hypercholesterolemia, He= Heterozygous, Ho= Homozygous, LDL= low density lipoprotein.

In this study, the proportion of individuals with a recorded diagnosis of FH and who were deemed to potentially have FH using an algorithm that utilized LDL-C measurement and other parameters was higher after the age of 40. This is contrary to what is desirable when screening for a condition which exposes an individual to high cholesterol levels from birth. Population attributable risk is used to estimate the proportion of events that could be reduced if that condition were eliminated. Among individuals in our study with elevated LDL-C, over half of premature mortality was attributed to the FH_Potential_ cohort. Within the limitations of study design, our observations could have other practical implications. For example, identification of potential cases of FH through the systematic application of a routine diagnostic algorithm may offer a simple tool to screen individuals prior to more formal assessment such as genetic testing. The DLCN algorithm we applied to classify FH_Potential_ individuals in this study has been shown to predict the presence of FH mutations, with odds ratios for a mutation of 439 and 90 for Definite and Probable scores, respectively [25]. The potential yield from this approach would further augment cascade testing from index cases, currently used in the UK, and help to increase the overall low detection rates for this condition. Furthermore, irrespective of whether a pathogenic mutation was present among the FH_Potential_ group, these individuals represent a high-risk group that may also benefit from early lifestyle changes and risk factor modification [26,27]. Finally, given our findings and the declining costs of biochemical tests, universal cholesterol screening (for instance, by the age of 20 years) may yield greater diagnostic returns in FH detection than current approaches and potentially reduce the premature burden of cardiovascular disease in this population [11] (Fig. 4).

The strengths and limitations of our study merit careful consideration. A major strength is the large size and granularity of the CPRD database including risk factor and prescription codes [12], its geographical coverage and thus generalizability, and linkage to mortality and hospital episode statistics. The widely accepted validity of the diagnostic algorithms [28,29] implemented to categorize the FH_Potential_ cohort, and the reliance on elevated LDL-C to achieve the 'Potential' threshold, gives credence to our classification. Our diagnostic classification was further validated via the restriction to the post mid-2008 timeline (introduction date of NICE guidance on FH-diagnosis). Laboratory test results may fluctuate and tend to regress to the mean; hence, had we opted to rely on limited information pre-2008 (for example, a single LDL-C test result), we would likely have introduced bias. For mortality, we only included patients following an LDL-C measurement, regardless of when that happened (pre-2008 or post-2008). We included time after the LDL-C measurement because individuals could not have died before they had their LDL-C test. Immortal time bias was not a confounder, since for all mortality analyses we included time following the first LDL-C measure, regardless of whether it occurred pre- or post-2008 (this also ensured that person-time would not be included for a period when patients could not have died; the period prior to having their LDL-C record).

### 3.1 Limitations

As with all studies using electronic medical records, the data captured may not be systematic and thus prone to bias. Specifically, information on parameters which make up the Dutch Lipid Network Criteria score, of which LDL-C is a key component, may be absent. A key limitation of our study was the potential under-underestimation of cardiovascular events and mortality, since the diagnosis of FH requires a blood test, and in the absence of universal screening many patients lacked LDL-C measurements and, hence, were not captured in this analysis. Furthermore the gold standard genetic diagnosis was not available in GP records. Our design has practical limitations and though many of the FH_Potential_ cases are likely to truly have had FH some may not. In that regard, we sacrificed specificity for sensitivity and we should acknowledge that perhaps beyond our initial aims these findings show the risk from high LDL-C per se. Moreover, a survival bias may exist for both the FH_Potential_ and FH_Coded_ groups as the sickest patients may have died before cholesterol levels were recorded or a diagnosis was achieved. Furthermore, patients in the FH_Coded_ group may have had their diagnosis, initial LDL-C measurements and initiation/changes in therapy made in tertiary care. Information from this encounter may not have been transcribed into the CPRD record, and thus be missing in our analysis, which may account for the low baseline use of statin. The observational nature of our study cannot entirely exclude the possibility of residual confounding, as a possible alternative explanation for our findings. Although we observed worse outcomes among individuals potentially with FH and no recorded diagnosis vs those with a recorded diagnosis, by design our study cannot elucidate the reasons behind this, nor directly infer causality that making a diagnosis would translate into better outcomes; such conclusions would require specific trials.

Finally, our study cannot provide estimates of prevalence of FH. By definition in order to be included, individuals needed a measurement of LDL-C or a coded diagnosis of FH out of all available records (5,677,532). Hence combining FH_Coded_ and FH_Potential_ appears to have a prevalence exceeding the 1:311 reported in systematic reviews as the denominator reflects inclusion criteria for this study rather than the whole population (CPRD dataset) from which this cohort is derived (1,729,046). Similarly, LDL-C measurements contribute to the denominator making up FH_Potential_ or FH_Unlikely_, which is different to the whole CPRD dataset which includes those without LDL-C measurements. Moreover, those coded as FH may have been coded incorrectly as many also had higher triglyceride levels than might be expected. Further validation of the findings of the present study in other large real world datasets are desirable but may not be easily feasible as ICD codes for FH are not standard practice in many parts of the world or have only recently been implemented..

## 4 Conclusion

Recorded diagnoses of FH before the age of 40 are low and in the general population LDL-C is largely measured after the age of 40. Compared to individuals with a recorded diagnosis of FH, those individuals potentially having FH but in whom a diagnosis had not been recorded appear to have worse health outcomes, including loss of up to 16 life years by the age of 18. Such individuals account for more than half of all premature deaths among those with hypercholesterolemia. Limitations of the study design notwithstanding, our findings further strengthen the case for screening for FH earlier in life.

## Sources of funding

Amgen-sponsored study. Amgen Ltd provided epidemiological and statistical support analysing the CPRD data in line with the design of the analysis plan by the first and last authors (KKR and JA). Authors employed by the sponsor contributed to the interpretation of the data. The final decision to submit the manuscript lay with the first author KKR.

## CRediT authorship contribution statement

**Kausik K. Ray:** Conceptualization, Funding acquisition, Methodology, Supervision, Writing – original draft, Writing – review & editing. **Demetris Pillas:** Data curation, Formal analysis, Software. **Savvas Hadjiphilippou:** Writing – original draft, Writing – review & editing. **Kamlesh Khunti:** Writing – original draft, Writing – review & editing. **Sreenivasa Rao Kondapally Seshasai:** Writing – review & editing. **Antonio J. Vallejo-Vaz:** Writing – review & editing. **David Neasham:** Data curation, Formal analysis, Software. **Janet Addison:** Data curation, Formal analysis, Writing – original draft, Writing – review & editing.

## Declaration of Competing Interest

Professor Kausik K. Ray reports the following; Unrestricted research grants to Imperial College London from Amgen, Daiichi Sankyo, Regeneron, Sanofi, SC, EC or advisory boards honoraria from Novartis, Esperion, Daiichi Sankyo, Abbott, Bayer, Eli Lilly, Silence Therapeutics, CSL Behring, New Amsterdam Pharma, Sanofi, Amgen, Novo Nordisk, BI, Scribe, Vaxxinity, CRISPR, AZ, Kowa, Cargene, Honoria for CME and non CME from Novartis, Novo Nordisk, BI, AZ, Viatris, Daiichi Sankyo, Amgen, Sanofi and stock options PEMI-31.

Dr. Demetris Pillas provided consultancy services to Amgen Ltd.

Dr. Savvas Hadjiphilippou has no disclosures.

Professor Kamlesh Khunti has received research grants from Lilly, Sanofi-Aventis, Boehringer Ingelheim, Merck, Sharpe & Dohme, and Novo Nordisk, has provided consultancy services to Amgen, Novartis, Novo Nordisk, Sanofi-Aventis, Lilly, Servier, and Merck, Sharpe & Dohme, has served in non-remunerative positions of influence at Lilly, Sanofi-Aventis, Merck, Sharpe & Dohme, and Novo Nordisk and has participated in Speakers Bureau for Lilly, Sanofi-Aventis, Merck, Sharpe & Dohme, and Novo Nordisk.

Dr. Sreenivasa Rao Kondapally Seshasai has provided consultancy services to Amgen.

Dr. Antonio J. Vallejo-Vaz reports current or past participation in research grants to Imperial College London from Pfizer, Amgen, MSD, Sanofi-Aventis, Daiichi Sankyo, and Regeneron; personal fees for consulting from Bayer and Regeneron; and honoraria for lectures from Amgen, Mylan, Akcea, and Ferrer; all outside the submitted work.

Dr David Neasham is employed at Amgen Ltd.

Ms. Janet Addison was previously employed at Amgen Ltd.

## Appendix Supplementary materials

Image, application 1
