# Supplementary material for: Premature morbidity and mortality associated with potentially undiagnosed familial hypercholesterolemia in the general population
Source: Am J Prev Cardiol. 2023 Sep 6;15:100580. doi: 10.1016/j.ajpc.2023.100580 (PMC10506055; doi:10.1016/j.ajpc.2023.100580)
Supplement: Supplementary file 1 [file mmc1.docx]

Supplemental Appendix

This appendix has been provided by the authors to give readers additional information about their work.

Supplement to: Ray, K. K., Pillas, D., Hadjiphilippou, S., et al. ***Premature Morbidity and Mortality Associated with Potentially Undiagnosed Familial Hypercholesterolemia in the General Population***

**Table of Contents**

[List of Investigators 4](#_Toc71612563)

[Supplemental Figures 5](#_Toc71612564)

[Supplemental Tables 8](#_Toc71612565)

[Supplemental Methods 20](#_Toc71612566)

[References 90](#_Toc71612570)

**List of Supplemental Figures**

[Supplemental Figure 1. Patient Flow Chart 5](#_Toc71611532)

[Supplemental Figure 2. Life Expectancy by Age in the FH_Coded_, FH_Potential_, and FH_Unlikely_ Groups. 7](#_Toc71611533)

**List of Supplemental Tables**

[Supplemental Table 1. Proportion of People Identified as FH_Coded_ and FH_Potential_ (per 1000) within this study design. 8](#_Toc141451232)

[Supplemental Table 2. Key parameters leading to consideration as FH_Potential_ using DLCN or EUROASPIRE criteria. 9](#_Toc141451233)

[Supplemental Table 3. Proportion of FH potential population considered probable or definite FH by DLCN or EUROASPIRE criteria. 9](#_Toc141451234)

[Supplemental Table 4. Incidence rate per 1000 person years by age groups. 10](#_Toc141451235)

[Supplemental Table 5. Sensitivity Analyses: Incidence Rates and Incidence Rate Ratios of a Premature Cardiovascular Event (After Excluding Individuals With Diabetes, Hypertension, or who are Smokers). 11](#_Toc141451236)

[Supplemental Table 6. Premature cardiovascular events occurring in FH_Potential_ categories (Probable or Definite) compared to FH_Unlikely_. 12](#_Toc141451237)

[Supplemental Table 7. Premature mortality occurring in FH_Potential_ categories (Probable or Definite) compared to FH_Coded._ 13](#_Toc141451238)

[Supplemental Table 8. Population-Attributable Risk Analysis. 14](#_Toc141451239)

[Supplemental Table 9. Demographic and Clinical Characteristics of study population, at baseline before exclusion of those with prior ASCVD. 15](#_Toc141451240)

[Supplemental Table 10. Proportion of FH coded population considered probable or definite FH by DLCN or EUROASPIRE criteria. 18](#_Toc141451241)

[Supplemental Table 11. Proportion of FH potential or FH coded population according to LDL-C level and treatment with Lipid Modifying Therapy (LMT). 18](#_Toc141451242)

**List of Supplemental Methods Tables**

[Supplemental Methods Table 1. CPRD Read Codes Used for Definition of FH, With Corresponding Labels. 21](#_Toc141452162)

[Supplemental Methods Table 2. Algorithm of Dutch Lipid Clinic Network (DLCN) criteria, as Applied to Data Available in CPRD. 22](#_Toc141452163)

[Supplemental Methods Table 3. Algorithm of EUROASPIRE, as Applied to Data Available in CPRD. 25](#_Toc141452164)

[Supplemental Methods Table 4. CPRD Read / Medical Codes (MED Code) Used for Definitions/Outcomes Relating to DLCN/EUROASPIRE Criteria, With Corresponding Labels. 27](#_Toc141452165)

[Supplemental Methods Table 5. Description of study variables, associated data sources and method of assessment. 71](#_Toc141452166)

[Supplemental Methods Table 6. Causes of death included under ‘mortality attributable to circulatory causes’. 72](#_Toc141452167)

# List of Investigators

Kausik K. Ray, FRCP, Demetris Pillas, Ph.D., Kamlesh Khunti, FMedSci., Sreenivasa Rao Kondapally Seshasai, MRCP, Savvas Hadjiphilippou, M.B.B.S., Antonio J. Vallejo-Vaz, Ph.D., David Neasham, Ph.D., Janet Addison, M.Sc.

# Supplemental Figures

**Supplemental Figure 1. Patient Flow Chart**

| Adult (≥18 years of age) patients registered in the CPRD in up-to-standard GP practices within the 5-year period 1/8/2008 to 31/7/2013  n = 5,677,532 |
| --- |

**Excluded Patients**

Patients with:

1) neither an FH code nor an LDL-C value recorded in CPRD

n = 3,786,255

2) with a prior history of cardiovascular disease at baseline

n = 162,231

n = 3,948,486

| **FH_Coded_**  Patients with an FH diagnostic code recorded in CPRD  n = 6,843 | **FH_Potential_**  Patients with at least one LDL-C value and not coded as FH but identified as possibly having FH based on achieving a pre-defined ‘Definite’ or ‘Probable’ score calculated according to either the Dutch Lipid Clinic Network (DLCN) criteria^1^ or published EUROASPIRE criteria^2^  n= 13,459 | **FH_Unlikely_**  Patients with at least one LDL-C value, who do not meet the criteria for Coded FH or Potential FH  n= 1,708,744 |
| --- | --- | --- |

All patients had the opportunity of a minimum 12-month follow-up period from baseline to the end of study observation on 31/7/2014. Patients did not move between cohorts.

CPRD= Clinical Practice Research Datalink CPRD, FH= Familial Hypercholesterolemia, LDL-C= low density lipoprotein- C.

**Supplemental Figure 2.** **Life Expectancy by Age in the FH_Coded_, FH_Potential_, and FH_Unlikely_ Groups.**


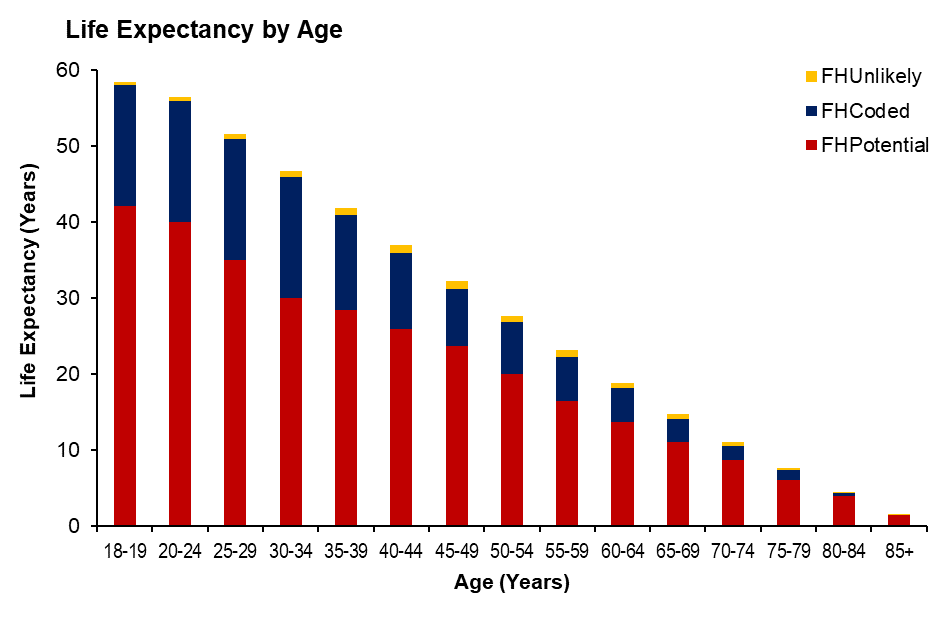


FH= Familial Hypercholesterolemia

# Supplemental Tables

**Supplemental Table 1. Proportion of People Identified as FH_Coded_ and FH_Potential_ (per 1000) within this study design.**

|  | **FH_Coded_** | **FH_Potential_** |
| --- | --- | --- |
|  | n, rate (95% CIs) | n, rate (95% CIs) |
|  |  |  |
| **All Patients** | 6843, 1.3 (1.2–1.3) | 13459, 7.8 (7.7–7.9) |
|  |  |  |
| **Age** |  |  |
| 18–39 y | 703, 0.3 (0.3–0.4) | 271, 1.8 (1.6–2.0) |
| 40–59 y | 2848, 1.6 (1.5–1.6) | 4046, 6.3 (6.1–6.5) |
| 60+ y | 3292, 2.1 (2.1–2.2) | 9142, 9.8 (9.6–10.0) |
|  |  |  |
| **Gender** |  |  |
| Female | 3753, 1.3 (1.3–1.4) | 7818, 8.7 (8.5–8.9) |
| Male | 3090, 1.2 (1.1–1.2) | 5641, 6.8 (6.7–7.0) |
|  |  |  |
| **Ethnicity** |  |  |
| White | 3550, 2.1 (2.0–2.2) | 4284, 7.9 (7.6–8.1) |
| Non-white | 854, 0.8 (0.7–0.9) | 2494, 7.3 (7.1–7.6) |
| Unknown | 2439, 0.9 (0.9–0.9) | 6681, 8.0 (7.8–8.2) |
|  |  |  |
| **Region** |  |  |
| England | 4947, 1.1 (1.1–1.2) | 10250, 7.3 (7.2–7.4) |
| Scotland | 480, 0.9 (0.8–1.0) | 902, 10.4 (9.7–11.1) |
| Wales | 1325, 3.0 (2.8–3.1) | 1587, 9.4 (9.0–9.9) |
| Northern Ireland | 91, 0.6 (0.5–0.8) | 720, 11.3 (10.5–12.1) |

CI= confidence interval, FH= familial hypercholesterolemia, y= years.

**Supplemental Table 2. Key parameters leading to consideration as FH_Potential_ using DLCN or EUROASPIRE criteria.**

| **Key Event Category / Total FH-Potential (N=13459)** | **DLCN (N=2873)** | **EUROASPIRE (N=10586)** |
| --- | --- | --- |
| 1) High LDL-C | 2064 (71.8%) | 10114 (95.5%) |
| 2) Family History | 754 (26.2%) | 472 (4.5%) |
| 3) Physical Exam | 57 (2.0%) | n / a |
| 4) Genetic Testing | 0 (0.0%) | n / a |

DLCN= Dutch Lipid Clinic Network, FH= familial hypercholesterolemia, LDL-C= low density lipoprotein-C.

**Supplemental Table 3. Proportion of FH potential population considered probable or definite FH by DLCN or EUROASPIRE criteria.**

| **Population / Total FH-Potential (N=13459)** | **DLCN (N=2873)** | **EUROASPIRE (N=10586)** |
| --- | --- | --- |
| Probable | 2701 (94) | 9776 (92.3) |
| Definite | 172 (6) | 810 (7.7) |

DLCN= Dutch Lipid Clinic Network, LDL-C= low density lipoprotein-C.

**Supplemental Table 4. Incidence rate per 1000 person years by age groups.**

| **Age (y)** | **FH_Coded_** | | | **FH_Potential_** | | | **FH_Unlikely_** | | |
| --- | --- | --- | --- | --- | --- | --- | --- | --- | --- |
|  | All | Female | Male | All | Female | Male | All | Female | Male |
| 18-24 | 0.53 |  | 1.07 |  |  |  | 0.09 | 0.1 | 0.09 |
| 25-29 | 1.1 | 0.73 | 1.47 |  |  |  | 0.15 | 0.14 | 0.15 |
| 30-34 | 0.87 | 1.38 | 0.41 | 0.28 | 0.73 |  | 0.2 | 0.17 | 0.23 |
| 35-39 | 2.02 | 0.33 | 3.35 | 0.81 | 0.66 | 0.92 | 0.31 | 0.22 | 0.41 |
| 40-44 | 2.6 | 1.88 | 3.21 | 1.86 | 1.4 | 2.24 | 0.62 | 0.42 | 0.84 |
| 45-49 | 6.24 | 3.65 | 8.75 | 3.29 | 1.95 | 4.57 | 1.15 | 0.76 | 1.56 |
| 50-54 | 7.22 | 4.65 | 10.29 | 5.36 | 3.34 | 7.84 | 1.86 | 1.18 | 2.55 |
| 55-59 | 8.71 | 4.96 | 14.45 | 6.92 | 4.01 | 11.46 | 2.68 | 1.58 | 3.82 |
| 60-64 | 11.08 | 8.39 | 16.15 | 7.68 | 6.1 | 10.66 | 3.96 | 2.53 | 5.52 |
| 65-69 | 16.99 | 11.69 | 28.83 | 10.33 | 7.9 | 15.66 | 5.81 | 3.89 | 8.06 |
| 70-74 | 18.22 | 16.35 | 23.05 | 12.91 | 10.35 | 19.47 | 8.06 | 5.8 | 10.96 |
| 75-79 | 19.9 | 16.32 | 30.13 | 19.59 | 16.95 | 28.03 | 12.25 | 9.57 | 16.12 |
| 80-84 | 26.55 | 25.74 | 29.18 | 26.4 | 26.44 | 26.28 | 19.12 | 15.84 | 24.68 |
| 85-89 | 43.87 | 38.8 | 59.38 | 37.62 | 38.1 | 35.46 | 30.07 | 26.61 | 37.19 |
| 90+ | 49.83 | 64.94 |  | 73.31 | 79.19 | 28.65 | 61.46 | 57.96 | 70.67 |

FH= familial hypercholesterolemia, y= years.

**Supplemental Table 5. Sensitivity Analyses:** **Incidence Rates and Incidence Rate Ratios of a Premature Cardiovascular Event (After Excluding Individuals With Diabetes, Hypertension, or who are Smokers).**

|  | **Original Result** | **Sensitivity Analysis I** | **Sensitivity Analysis II** |
| --- | --- | --- | --- |
|  |  | **Excluding Diabetes and Hypertension** | **Excluding Diabetes, Hypertension, and Smoking** |
|  | **Premature Cardiovascular Incidence Rate**  **Incidence Rate (95% CIs)** | **Premature Cardiovascular Incidence Rate**  **Incidence Rate (95% CIs)** | **Premature Cardiovascular Incidence Rate**  **Incidence Rate (95% CIs)** |
| **Premature Cardiovascular Event** |  |  |  |
| FH_Coded_ | 0.0042 (0.0037, 0.0048) | 0.0022 (0.0019, 0.0026) | 0.0017 (0.0014, 0.0021) |
| FH_Potential_ | 0.0034 (0.0030, 0.0038) | 0.0016 (0.0014, 0.0019) | 0.0011 (0.0009, 0.0014) |
| FH_Unlikely_ | 0.0010 (0.0010, 0.0010) | 0.0006 (0.0006, 0.0006) | 0.0005 (0.0005, 0.0005) |
| Incidence Rate Ratio (FH_Potential_ vs FH_Coded_) | 0.81 (0.68, 0.96), p=0.017 | 0.75 (0.60, 0.95), p=0.017 | 0.62 (0.45, 0.86), p=0.005 |
| Incidence Rate Ratio (FH_Potential_ vs FH_Unlikely_) | 3.41 (3.04, 3.84), p<0.001 | 2.61 (2.24, 3.07), p<0.001 | 1.95 (1.53, 2.48), p<0.001 |

Sensitivity analysis excluded patients with a history of diabetes and/or hypertension, and then current smoking. FH= familial hypercholesterolemia.

**Supplemental Table 6. Premature cardiovascular events occurring in FH_Potential_ categories (Probable or Definite) compared to FH_Unlikely_**.

| **Cohort** | **FH_Unlikely_** | **FH_Potential_** | | | | | |
| --- | --- | --- | --- | --- | --- | --- | --- |
|  |  | **Probable** | | | **Definite** | | |
|  | **N (%)** | **N (%)** | **Hazard ratio (95% CI) vs FH_Unlikely_** | **p** | **N (%)** | **Hazard ratio (95% CI) vs FH_unlikely_** | **P** |
| **Overall** | 11469/ 1211283 (0.9) | 225/ 8184  (2.7) | 3.08  (2.70, 3.52) | <0.001 | 59/969  (6.1) | 6.50  (5.03, 8.39) | <0.001 |
| **Men** | 6312/ 556641  (1.1) | 124/ 3493  (3.5) | 3.16  (2.65, 3.78) | <0.001 | 24/424  (5.7) | 5.12  (3.43, 7.65) | <0.001 |
| **Women** | 5157/ 654642  (0.8) | 101/ 4691  (2.2) | 3.02  (2.48, 3.68) | <0.001 | 35/545  (6.4) | 8.21  (5.89, 11.45) | <0.001 |

**Supplemental Table 7. Premature mortality occurring in FH_Potential_ categories (Probable or Definite) compared to FH_Coded_**_._

| **Cohort** | **FH_Coded_** | **FH_Potential_** | | | | | |
| --- | --- | --- | --- | --- | --- | --- | --- |
|  |  | **Probable** | | | **Definite** | | |
|  | **N (%)** | **N (%)** | **Hazard ratio (95% CI) vs FH_Coded_** | **p** | **N (%)** | **Hazard ratio (95% CI) vs FH_Coded_** | **P** |
| **Overall** | 16/5560  (0.3) | 65/8184  (0.8) | 2.99  (1.73-5.16) | <0.001 | 10/969  (1.0) | 3.71  (1.68-8.18) | <0.001 |
| **Men** | 7/2572  (0.3) | 27/3493  (0.8) | 2.99  (1.30-6.86) | 0.01 | 3/424  (0.7) | 2.73  (0.70-10.57) | 0.14 |
| **Women** | 9/2988  (0.3) | 38/4691  (0.8) | 3.01  (1.45-6.22) | 0.003 | 7/545  (1.3) | 4.22  (1.57-11.34) | 0.004 |

**Supplemental Table 8.** **Population-Attributable Risk Analysis.**

|  | **Population Attributable Risk %** | |
| --- | --- | --- |
|  | All patients included | Exclusion of patients with a history of hypertension, diabetes and smokers at baseline |
| Premature mortality | 67.53 | 55.58 |
|  |  |  |

The percentage of population attributable risk represents the percent of disease incidence/risk of premature death in the combined FH_Coded_/FH_Potential_ patient populations that would be eliminated if the exposure FH_Potential_ were eliminated.

**Supplemental Table 9. Demographic and Clinical Characteristics of study population, at baseline before exclusion of those with prior ASCVD.**

|  |  |  |  |
| --- | --- | --- | --- |
|  | FH_Coded_ | FH_Potential_ | FH_Unlikely_ |
|  | n (%) | n (%) | n (%) |
|  | n = 7193 | n = 21627 | n = 1862457 |
| **Demographic Characteristics** |  |  |  |
| Age, years, mean (SD) | 51.4 (13.8) | 61.0 (11.6) | 63.6 (16.2) |
| Gender |  |  |  |
| Male | 3274 (45.5) | 9806 (45.3) | 906590 (48.7) |
| Female | 3919 (54.5) | 11821 (54.7) | 955860 (51.3) |
| Missing/unknown | 0 (0.0) | 0 (0.0) | 7 (0.0) |
| Ethnicity |  |  |  |
| White | 3701 (51.5) | 6937 (32.1) | 584797 (31.4) |
| Non-white | 880 (12.2) | 3863 (17.9) | 359003 (19.3) |
| Missing/unknown | 2612 (36.3) | 10827 (50.1) | 918657 (49.3) |
| Region |  |  |  |
| England | 5139 (71.4) | 16537 (76.5) | 1518046 (81.5) |
| Scotland | 500 (7.0) | 1515 (7.0) | 94952 (5.1) |
| Wales | 1455 (20.2) | 2467 (11.4) | 181143 (9.7) |
| Northern Ireland | 99 (1.4) | 1108 (5.1) | 68316 (3.7) |
| Missing/unknown | 0 (0.0) | 0 (0.0) | 0 (0.0) |
| **Clinical Characteristics** |  |  |  |
| BMI |  |  |  |
| Normal weight (BMI: <25) | 1096 (15.2) | 3862 (17.9) | 439536 (23.6) |
| Overweight (BMI: 25-29.9) | 1583 (22.0) | 7420 (34.3) | 532643 (28.6) |
| Obese (BMI: 30+) | 1041 (14.5) | 6906 (31.9) | 471985 (25.3) |
| Missing/unknown | 3473 (48.3) | 3439 (15.9) | 418293 (22.5) |
| Smoking |  |  |  |
| Never smoked | 2314 (32.2) | 8273 (38.3) | 851756 (45.7) |
| Previous smoker | 1066 (14.8) | 6320 (29.2) | 522588 (28.1) |
| Current smoker | 969 (13.5) | 5595 (25.9) | 257350 (13.8) |
| Missing/unknown | 2844 (39.5) | 1439 (6.7) | 230763 (12.4) |
| Diabetes | 148 (2.1) | 3251 (15.0) | 186967 (10.0) |
| Hypertension | 937 (13.0) | 7093 (32.8) | 338253 (18.2) |
| Previous CVD history |  |  |  |
| Acute Myocardial Infarction | 88 (1.2) | 2745 (12.7) | 53645 (2.9) |
| Peripheral Arterial Disease | 7 (0.1) | 287 (1.3) | 16993 (0.9) |
| Ischaemic Stroke | 45 (0.6) | 825 (3.8) | 50581 (2.7) |
| Transient Ischemic Attack | 42 (0.6) | 832 (3.8) | 37101 (2.0) |
| Unstable Angina | 223 (3.1) | 5616 (26.0) | 114710 (6.2) |
| Coronary Revascularization | 56 (0.8) | 1747 (8.1) | 39496 (2.1) |
| Any previous CVD history | 350 (4.9) | 8168 (37.8) | 216031 (11.6) |
| Number of previous CV events |  |  |  |
| Mean (SD) | 0.1 (1.1) | 1.3 (3.1) | 0.4 (2.3) |
| 1 event | 159 (2.2) | 3117 (14.4) | 78782 (4.2) |
| 2 events | 71 (1.0) | 1503 (6.8) | 43565 (2.3) |
| 3 events | 40 (0.6) | 913 (4.2) | 26236 (1.4) |
| >3 events | 80 (1.1) | 2635 (12.2) | 67448 (3.6) |

ASCVD= Atherosclerotic Cardiovascular Disease, BMI= body-mass index, CVD= Cardiovascular Disease, FH= familial hypercholesterolemia, HDL= high density lipoprotein.

**Supplemental Table 10.** **Proportion of FH coded population considered probable or definite FH by DLCN or EUROASPIRE criteria.**

| **Population / Total FH-Coded (N=** **6843)** | **DLCN**  **(N=1711)** | **EUROASPIRE (N=5132)** |
| --- | --- | --- |
| Probable | 1536 (89.8) | 4563 (88.9) |
| Definite | 175 (10.2) | 569 (11.1) |

DLCN= Dutch Lipid Clinic Network, LDL-C= low density lipoprotein-C.

**Supplemental Table 11. Proportion of FH potential or FH coded population according to LDL-C level and treatment with Lipid Modifying Therapy (LMT).**

| **Population – Total FH-Potential or FH Coded** | **Treated with LMT & LDL-C >309.4mg/dL** | **Untreated with LMT & LDL-C >502.7mg/dL** |
| --- | --- | --- |
| FH potential (N=13459) | 48 (0.36%) | 0 (0.0) |
| FH coded (N=6843) | 11 (0.16%) | 0 (0.0) |

LMT= Lipid Modifying Therapy, LDL-C= low density lipoprotein-C.

# Supplemental Methods

## Estimation of life expectancy

We employed the method used by the UK Office of National Statistics (ONS) to calculate life expectancy by 5-year age bands and 95 per cent confidence intervals for sub-national areas in the UK. This entailed using an ONS-provided template to construct separate Life Tables for each patient group that is based on the revised Chiang method (Chiang II) (1).

## Estimation of population attributable risk percentage (PAR%)

PAR% was defined as the proportion of disease incidence (or risk) in a given population (i.e. one that includes both exposed and non-exposed) that is due to the exposure. Therefore, it represents the percentage of disease incidence/risk (in the population) that would be eliminated if the exposure were eliminated.

In the case of this study, PAR% represents the percent of disease incidence/risk of premature death in the combined FH_Coded_/FH_Potential_ patient populations, that would be eliminated if the exposure (i.e. non-diagnosis in the FH_Potential_) were eliminated.

## ICD-10 and OPCS codes used in HES data for definitions/outcomes relating to DLCN/EUROASPIRE criteria

**CHD**

ICD-10 codes I20.0 to I25.99

OPCS codes K40.0 to K46.99, K49.0 to K50.99, K75.0 to K75.99

**CVD-PVD**

ICD-10 codes I60.0 to I69.99 or I70.0 to I70.99 or I73.00 to I73.99 or G45.8 or G45.9

**EUROASPIRE Coronary Event**

ICD-10 codes I21.0 to I21.99

**Tendon Xanthoma**

ICD-10 code E75.5

**Corneal Arcus**

ICD-10 code H18.4

**Supplemental Methods Table 1. CPRD Read Codes Used for Definition of FH, With Corresponding Labels.**

| **Read Code** | **Label** |
| --- | --- |
| C3200. | Familial hypercholesterolemia |
| C3201. | Hyperbetalipoproteinemia |
| C3203. | Low-density-lipoprotein-type (LDL) hyperlipoproteinemia |
| C3204. | Fredrickson’s hyperlipoproteinemia, type Iia |
| C3205. | Familial defective apolipoprotein B-100 |
| C3220. | Familial combined hyperlipidemia |

Source: These Read codes were used to identify individuals with FH in the Health and Social Care Information Centre - Quality and Outcome Framework (HSCIC-QOF) Cardiovascular Disease Primary Prevention (CVD-PP) Indicator Set. HSCIC. 2014 (2).

The CPRD record for each FH_Coded_ patient was searched for evidence of an LDL cholesterol measurement and/or a routine health check and/or a cardiovascular event within the 12 months prior to the date of entry of the coded diagnosis. None were found for approximately 50% of the FH_Coded_ patients; this apparent absence of clinical investigation preceding diagnosis may indicate that diagnosis was made outside primary care.

CPRD= Clinical Practice Research Datalink, FH= Familial hypercholesterolemia.

**Supplemental Methods Table 2. Algorithm of Dutch Lipid Clinic Network (DLCN) criteria, as Applied to Data Available in CPRD.**

| **Criteria** | **Points** | **Availability in CPRD** | **CPRD Read / MEDcodes (with labels/descriptions)** |
| --- | --- | --- | --- |
| **Group 1: Family History**  First-degree relative with premature coronary heart disease [< age 55 (men) / 60 (women)]  First-degree relative with LDL cholesterol > 95^th^ percentile by age, gender for country  First-degree relative with tendon xanthoma and/or corneal arcus  OR  Children aged <18 years with LDL cholesterol > 95^th^ percentile by age, gender for country | 1  1  2  2 | Available  Partly available  Not available  Not available | Available in CPRD code list under ‘First-degree relative with premature CHD/CVD’  1262.11 (FH: Cholesterol high), 1262.00 (FH: Raised blood lipids), 126B.00 (FH: Hypercholesterolemia in first degree relative), 1269.00 (Family history of FH)  Not Available  Not Available |
| **Group 2: Personal Clinical History**  Premature coronary heart disease [< age 55 (men) / 60 (women)]  Premature cerebrovascular or peripheral vascular disease [< age 55 (men) / 60 (women)] | 2  1 | Available  Available | Definition of CHD (Available in CPRD code list under CHD)  Definition of cerebrovascular & peripheral vascular disease (Available in CPRD code list under ‘CVD-PVD’) |
| **Group 3: Physical Exam**  Tendon xanthoma  Corneal arcus (arcus senilis) in subject aged <45 years | 6  4 | Available  Available | N228.00 (Tendinous Xanthoma)  F4B4100, 22E2.11 (Arcus Senilis) |
| **Group 4: LDL Cholesterol Level**   1. >8.5 mmol/L 2. 6.5 - 8.4 mmol/L 3. 5.0 – 6.4 mmol/L 4. 4.0 – 4.9 mmol/L | 8  5  3  1 | Available | Based on enttype = 177 data2 status column (“LDL-C”); data1=3 and data3=96 |
| **Group 5: Genetic Testing**   1. Causative mutation in LDLR, ApoB, or PSK9 genes | 8 | Partly Available | C320500 (Familial defective apolipoprotein B-100) |

| Total score 6-8: Probable FH  Total score >8: Definite FH |
| --- |

CPRD= Clinical Practice Research Datalink, FH= Familial hypercholesterolemia, LDL= low-density lipoprotein

Source: Nordestgaard et al., 2013 (3)

**Supplemental Methods Table 3. Algorithm of EUROASPIRE, as Applied to Data Available in CPRD.**

| **Criteria** | **Points** | **Availability in CPRD** | **CPRD Medcodes** |
| --- | --- | --- | --- |
| **Group 1: Family History**   1. First-degree relative with premature cardiovascular disease [< age 55 (men) / 65 (women)] | 1 | Available | Available in CPRD code list under ‘First-degree relative with premature CHD/CVD’ |
| **Group 2: Personal Clinical History**   1. Age at the index coronary event was <55 / 60 years for men / women   OR  if self-reported age of first diagnosis of coronary heart disease was <55 / 60 years for men / women | 2 | Available | Definition of coronary event (Available in CPRD code list under ‘EUROASPIRE Coronary Event’)  Definition of CHD (Available in CPRD code list under ‘CHD’) |
| **Group 3: Untreated LDL Cholesterol (Corrected Scores)**   1. LDL cholesterol of 4.0-4.9 mmol/L 2. LDL cholesterol of 5.0-6.4 mmol/L 3. LDL cholesterol of 6.5-8.4 mmol/L 4. LDL cholesterol of 8.5 mmol/L or more | 1  3  5  8 | Available | Based on enttype = 177 data2 status column (“LDL-C”); data1=3 and data3=96.  (LDL-C score was corrected according to instructions / tables provided in Besseling, 2014 (4); De Backer, 2015 (5)) |

| Total score 6-8: Probable FH  Total score >8: Definite FH |
| --- |

CHD= coronary heart disease, CPRD= Clinical Practice Research Datalink, CVD= Cardiovascular disease, FH= Familial hypercholesterolemia, LDL= low-density lipoprotein.

Source: Besseling et al., 2014 (4); De Backer et al., 2015 (5)

**Supplemental Methods Table 4. CPRD Read / Medical Codes (MED Code) Used for Definitions/Outcomes Relating to DLCN/EUROASPIRE Criteria, With Corresponding Labels.**

| **READ Code** | **MED Code** | **Label** |
| --- | --- | --- |
| **First-Degree Relative With Premature CHD/CVD** | | |
| 12C2.00 | 3198 | IHD<60y |
| 12CI.00 | 28347 | Premature CHD |
| 12C2.13 | 12709 | Angina<60y |
| 12C2.12 | 12806 | Myocardial infarction <60y |
| 12C2.11 | 13270 | Myocardial infarction <60y |
| 12CP.00 | 18661 | Myocardial infarct in 1st degree male relative <55y |
| 12CN.00 | 19127 | Myocardial infarct in 1st degree female relative <65y |
| 12CM.00 | 26653 | Angina in 1st degree male relative <55y |
| 12CL.00 | 30789 | Angina in 1st degree female relative <65y |
| 12CV.00 | 96212 | Cardiovascular disease 1st degree male relative < 55y |
| 12CW.00 | 96596 | Cardiovascular disease 1st degree female relative < 65y |
| **CHD** | | |
| G30..12 | 2491 | Coronary thrombosis |
| G30..16 | 13571 | Thrombosis - coronary |
| G312.00 | 39449 | Coronary thrombosis not resulting in myocardial infarction |
| G311500 | 11983 | Acute coronary syndrome |
| 14AW.00 | 105216 | Acute coronary syndrome |
| 14A5.00 | 6336 | Angina pectoris |
| 14AJ.00 | 57062 | Angina in last year |
| 662K.00 | 13185 | Angina control |
| 662K000 | 19542 | Angina control |
| 662K100 | 15373 | Angina control |
| 662K200 | 14782 | Angina control |
| 662K300 | 29300 | Angina control |
| 662Kz00 | 15349 | Angina control |
| 8B27.00 | 45960 | Antianginal therapy |
| G33..00 | 1430 | Angina pectoris |
| G330.00 | 20095 | Angina decubitus |
| G330000 | 18125 | Nocturnal angina |
| G330z00 | 29902 | Angina decubitus |
| G331.00 | 12986 | Prinzmetal's angina |
| G331.11 | 11048 | Variant angina pectoris |
| G332.00 | 36854 | Coronary artery spasm |
| G33z.00 | 25842 | Angina pectoris |
| G33z000 | 66388 | Status anginosus |
| G33z100 | 54535 | Stenocardia |
| G33z200 | 7696 | Syncope anginosa |
| G33z300 | 1414 | Angina on effort |
| G33z400 | 32450 | Ischemic chest pain |
| G33z500 | 9555 | Post infarct angina |
| G33z600 | 26863 | New onset angina |
| G33z700 | 12804 | Stable angina |
| G33zz00 | 28554 | Angina pectoris |
| G311.00 | 36523 | Preinfarction syndrome |
| G311.11 | 4656 | Crescendo angina |
| G311.12 | 39655 | Impending infarction |
| G311.13 | 1431 | Unstable angina |
| G311.14 | 19655 | Angina at rest |
| G311000 | 61072 | Myocardial infarction aborted |
| G311011 | 55137 | MI - myocardial infarction aborted |
| G311100 | 7347 | Unstable angina |
| G311200 | 17307 | Angina at rest |
| G311300 | 34328 | Refractory angina |
| G311400 | 18118 | Worsening angina |
| G311z00 | 54251 | Preinfarction syndrome |
| G340.12 | 1344 | Coronary artery disease |
| P6y4.00 | 25481 | Coronary artery anomaly |
| P6y4z00 | 53546 | Coronary artery anomaly |
| G3…00 | 240 | Ischemic heart disease |
| G3z..00 | 1676 | Ischemic heart disease |
| G3…13 | 1792 | IHD - Ischemic heart disease |
| G31y.00 | 9413 | Other acute and subacute ischemic heart disease |
| G34z.00 | 15754 | Other chronic ischemic heart disease |
| G3y..00 | 22383 | Other specified ischemic heart disease |
| G31..00 | 27951 | Other acute and subacute ischemic heart disease |
| G31yz00 | 27977 | Other acute and subacute ischemic heart disease |
| G34..00 | 28138 | Other chronic ischemic heart disease |
| G34y.00 | 34633 | Other specified chronic ischemic heart disease |
| G34z000 | 18889 | Asymptomatic coronary heart disease |
| 14AL.00 | 45476 | Treatment for ischemic heart disease |
| Gyu3300 | 47637 | Other forms of chronic ischemic heart disease |
| Gyu3.00 | 52517 | Ischemic heart diseases |
| Gyu3200 | 68401 | Other forms of acute ischemic heart disease |
| 792..11 | 737 | Coronary artery bypass graft operations |
| 7920.00 | 18249 | Saphenous vein graft replacement of coronary artery |
| 7920.11 | 8312 | Saphenous vein graft bypass of coronary artery |
| 7920000 | 8679 | Saphenous vein graft replacement of one coronary artery |
| 7920100 | 7634 | Saphenous vein graft replacement of two coronary arteries |
| 7920200 | 7442 | Saphenous vein graft replacement of three coronary arteries |
| 7920300 | 11610 | Saphenous vein graft replacement of four+ coronary arteries |
| 7920y00 | 7137 | Saphenous vein graft replacement of coronary artery |
| 7920z00 | 51515 | Saphenous vein graft replacement coronary artery |
| 7921.00 | 9414 | Other autograft replacement of coronary artery |
| 7921.11 | 7134 | Other autograft bypass of coronary artery |
| 7921000 | 44561 | Autograft replacement of one coronary artery |
| 7921100 | 19413 | Autograft replacement of two coronary arteries |
| 7921200 | 10209 | Autograft replacement of three coronary arteries |
| 7921300 | 42708 | Autograft replacement of four of more coronary arteries |
| 7921y00 | 61310 | Other autograft replacement of coronary artery |
| 7921z00 | 7609 | Other autograft replacement of coronary artery |
| 7922.00 | 31556 | Allograft replacement of coronary artery |
| 7922.11 | 32651 | Allograft bypass of coronary artery |
| 7922000 | 70111 | Allograft replacement of one coronary artery |
| 7922100 | 57241 | Allograft replacement of two coronary arteries |
| 7922200 | 45886 | Allograft replacement of three coronary arteries |
| 7922300 | 45370 | Allograft replacement of four or more coronary arteries |
| 7922y00 | 59423 | Other specified allograft replacement of coronary artery |
| 7922z00 | 48767 | Allograft replacement of coronary artery |
| 7923.00 | 19402 | Prosthetic replacement of coronary artery |
| 7923.11 | 36011 | Prosthetic bypass of coronary artery |
| 7923000 | 92419 | Prosthetic replacement of one coronary artery |
| 7923100 | 66664 | Prosthetic replacement of two coronary arteries |
| 7923200 | 66236 | Prosthetic replacement of three coronary arteries |
| 7923300 | 67761 | Prosthetic replacement of four or more coronary arteries |
| 7923z00 | 19193 | Prosthetic replacement of coronary artery |
| 7924.00 | 33461 | Revision of bypass for coronary artery |
| 7924000 | 52938 | Revision of bypass for one coronary artery |
| 7924100 | 67554 | Revision of bypass for two coronary arteries |
| 7924200 | 31540 | Revision of bypass for three coronary arteries |
| 7924300 | 101569 | Revision of bypass for four or more coronary arteries |
| 7924500 | 63153 | Revision of implantation of thoracic artery into heart |
| 7924y00 | 97953 | Other specified revision of bypass for coronary artery |
| 7924z00 | 57634 | Revision of bypass for coronary artery |
| 7925.00 | 37682 | Connection of mammary artery to coronary artery |
| 7925.11 | 28837 | Creation of bypass from mammary artery to coronary artery |
| 7925000 | 33718 | Double anastomosis of mammary arteries to coronary arteries |
| 7925011 | 48822 | LIMA sequential anastomosis |
| 7925012 | 92233 | RIMA sequential anastomosis |
| 7925100 | 31519 | Double implant of mammary arteries into coronary arteries |
| 7925200 | 44723 | Single anastomosis mammary art to left ant descend coronary art |
| 7925300 | 51507 | Single anastomosis of mammary artery to coronary artery |
| 7925311 | 22647 | LIMA single anastomosis |
| 7925312 | 68123 | RIMA single anastomosis |
| 7925400 | 68139 | Single implantation of mammary artery into coronary artery |
| 7925y00 | 37719 | Connection of mammary artery to coronary artery |
| 7925z00 | 56990 | Connection of mammary artery to coronary artery |
| 7926.00 | 96804 | Connection of other thoracic artery to coronary artery |
| 7926000 | 62608 | Double anastomosis thoracic arteries to coronary arteries |
| 7926200 | 67591 | Single anastomosis of thoracic artery to coronary artery |
| 7926300 | 60753 | Single implantation thoracic artery into coronary artery |
| 7926z00 | 72780 | Connection of other thoracic artery to coronary artery |
| 792C.00 | 55598 | Other replacement of coronary artery |
| 792C000 | 55092 | Replacement of coronary arteries using multiple methods |
| 792Cy00 | 93828 | Other specified replacement of coronary artery |
| 792Cz00 | 70755 | Replacement of coronary artery |
| 792D.00 | 34963 | Other bypass of coronary artery |
| 792Dy00 | 3159 | Other specified other bypass of coronary artery |
| 792Dz00 | 33471 | Other bypass of coronary artery |
| 792y.00 | 31571 | Other specified operations on coronary artery |
| 790H300 | 12743 | Revascularization of wall of heart |
| 7927500 | 5744 | Open angioplasty of coronary artery |
| 7928.00 | 2901 | Transluminal balloon angioplasty of coronary artery |
| 7928.11 | 5703 | Percutaneous balloon coronary angioplasty |
| 7928000 | 18670 | Percutaneous transluminal balloon angioplasty one coronary artery |
| 7928100 | 33735 | Percutaneous transluminal balloon angioplasty multiple coronary arteries |
| 7928200 | 42462 | Percutaneous transluminal balloon angioplasty bypass graft coronary artery |
| 7928300 | 86071 | Percutaneous transluminal cutting balloon angioplasty coronary artery |
| 7928y00 | 41547 | Transluminal balloon angioplasty of coronary artery |
| 7928z00 | 732 | Transluminal balloon angioplasty of coronary artery |
| 7929.00 | 24888 | Other therapeutic transluminal operations on coronary artery |
| 7929000 | 22828 | Percutaneous transluminal laser coronary angioplasty |
| 7929100 | 33650 | Percutaneous transluminal coronary thrombolysis with streptokinase |
| 7929111 | 40996 | Percutaneous transluminal coronary thrombolytic therapy- streptokinase |
| 7929200 | 66583 | Percutaneous transluminal injection therapy substitute to coronary artery |
| 7929300 | 19046 | Rotary blade coronary angioplasty |
| 7929400 | 8942 | Insertion of coronary artery stent |
| 7929500 | 42304 | Insertion of drug-eluting coronary artery stent |
| 7929600 | 93618 | Percutaneous transluminal atherectomy of coronary artery |
| 7929y00 | 6182 | Other therapeutic transluminal operation on coronary artery |
| 7929z00 | 31679 | Other therapeutic transluminal operation on coronary artery |
| 793G.00 | 43939 | Percutaneous transluminal balloon angioplasty stenting coronary artery |
| 793G000 | 60067 | Percutaneous transluminal ball angioplasty insert 1-2 drug stents coronary artery |
| 793G100 | 87849 | Percutaneous transluminal ball angioplasty ins 3 or more drug stents coronary artery |
| 793G200 | 85947 | Percutaneous transluminal balloon angioplasty insert 1-2 stents coronary artery |
| 793G300 | 92927 | Percutaneous coronary balloon angioplasty 3 more stents coronary artery |
| 793Gy00 | 96537 | Percutaneous transluminal balloon angioplasty stenting coronary artery |
| 793Gz00 | 61208 | Percutaneous transluminal balloon angioplasty stenting coronary artery |
| ZV45L00 | 6980 | Status following coronary angioplasty |
| **CVD-PVD** | | |
| G6z..00 | 10062 | Cerebrovascular disease |
| G6…00 | 2418 | Cerebrovascular disease |
| G671z00 | 12555 | Generalized ischemic cerebrovascular disease |
| G67..00 | 13577 | Other cerebrovascular disease |
| G68..00 | 23361 | Late effects of cerebrovascular disease |
| G67y.00 | 34117 | Other cerebrovascular disease |
| G67z.00 | 37493 | Other cerebrovascular disease |
| G671.00 | 40053 | Generalized ischemic cerebrovascular disease |
| 1477.00 | 41577 | Cerebrovascular disease |
| G68W.00 | 51138 | Sequelae/other and unspecified cerebrovascular diseases |
| G6y..00 | 51311 | Other specified cerebrovascular disease |
| F11x200 | 54744 | Cerebral degeneration due to cerebrovascular disease |
| Gyu6.00 | 73901 | Cerebrovascular diseases |
| Fyu5700 | 95347 | Other vascular syndromes / brain in cerebrovascular diseases |
| G679.00 | 98188 | Small vessel cerebrovascular disease |
| G73..00 | 5943 | Other peripheral vascular disease |
| G73..11 | 5702 | Peripheral ischemic vascular disease |
| G73..12 | 1826 | Ischemia of legs |
| G73..13 | 6827 | Peripheral ischemia |
| G734.00 | 105317 | Peripheral arterial disease |
| G73y.00 | 38907 | Other specified peripheral vascular disease |
| G73z.00 | 3530 | Peripheral vascular disease |
| G73z000 | 1517 | Intermittent claudication |
| G73z011 | 6853 | Claudication |
| G73z012 | 101866 | Vascular claudication |
| G73zz00 | 2760 | Peripheral vascular disease |
| Gyu7400 | 73961 | Other specified peripheral vascular diseases |
| G734.00 | 105317 | Peripheral arterial disease |
| G700.00 | 1318 | Aortic atherosclerosis |
| G340.00 | 5413 | Coronary atherosclerosis |
| G70..00 | 5640 | Atherosclerosis |
| G3…12 | 20416 | Atherosclerotic heart disease |
| G342.00 | 36609 | Atherosclerotic cardiovascular disease |
| G70y000 | 37199 | Carotid artery atherosclerosis |
| Gyu7000 | 100579 | Atherosclerosis of other arteries |
| G70y011 | 22677 | Carotid artery disease |
| G61..00 | 5051 | Intracerebral hemorrhage |
| G61..11 | 6960 | CVA - cerebrovascular acid due to intracerebral hemorrhage |
| G61..12 | 18604 | Stroke due to intracerebral hemorrhage |
| G610.00 | 31595 | Cortical hemorrhage |
| G611.00 | 40338 | Internal capsule hemorrhage |
| G612.00 | 46316 | Basal nucleus hemorrhage |
| G613.00 | 13564 | Cerebellar hemorrhage |
| G614.00 | 7912 | Pontine hemorrhage |
| G615.00 | 62342 | Bulbar hemorrhage |
| G616.00 | 30045 | External capsule hemorrhage |
| G617.00 | 30202 | Intracerebral hemorrhage, intraventricular |
| G618.00 | 57315 | Intracerebral hemorrhage, multiple localized |
| G61X.00 | 31060 | Intracerebral hemorrhage in hemisphere, unspecified |
| G61X000 | 28314 | Left sided intracerebral hemorrhage, unspecified |
| G61X100 | 19201 | Right sided intracerebral hemorrhage, unspecified |
| G61z.00 | 3535 | Intracerebral hemorrhage |
| G66..00 | 1469 | Stroke and cerebrovascular accident unspecified |
| G66..11 | 1298 | CVA unspecified |
| G66..12 | 6253 | Stroke unspecified |
| G66..13 | 6116 | CVA - Cerebrovascular accident unspecified |
| G660.00 | 18689 | Middle cerebral artery syndrome |
| G661.00 | 19280 | Anterior cerebral artery syndrome |
| G662.00 | 19260 | Posterior cerebral artery syndrome |
| G663.00 | 8443 | Brain stem stroke syndrome |
| G664.00 | 17322 | Cerebellar stroke syndrome |
| G667.00 | 7780 | Left sided CVA |
| G668.00 | 12833 | Right sided CVA |
| G669.00 | 16956 | Cerebral palsy, not congenital or infantile, acute |
| G6X..00 | 33543 | Cerebral infarction due / unspecified occlusion or stenosis/cerebral arteries |
| G63..00 | 45781 | Precerebral arterial occlusion |
| G63..11 | 57495 | Infarction - precerebral |
| G63..12 | 63830 | Stenosis of precerebral arteries |
| G630.00 | 32447 | Basilar artery occlusion |
| G631.00 | 4240 | Carotid artery occlusion |
| G631.11 | 2156 | Stenosis, carotid artery |
| G631.12 | 4152 | Thrombosis, carotid artery |
| G632.00 | 40847 | Vertebral artery occlusion |
| G633.00 | 98642 | Multiple and bilateral precerebral arterial occlusion |
| G634.00 | 2652 | Carotid artery stenosis |
| G63y.00 | 51326 | Other precerebral artery occlusion |
| G63z.00 | 71585 | Precerebral artery occlusion |
| G63y000 | 23671 | Cerebral infarct due to thrombosis of precerebral arteries |
| G63y100 | 24446 | Cerebral infarction due to embolism of precerebral arteries |
| G64..00 | 8837 | Cerebral arterial occlusion |
| G64..11 | 5363 | CVA - cerebral artery occlusion |
| G64..12 | 569 | Infarction - cerebral |
| G64..13 | 6155 | Stroke due to cerebral arterial occlusion |
| G640.00 | 16517 | Cerebral thrombosis |
| G640000 | 36717 | Cerebral infarction due to thrombosis of cerebral arteries |
| G641.00 | 15019 | Cerebral embolism |
| G641.11 | 34758 | Cerebral embolus |
| G641000 | 27975 | Cerebral infarction due to embolism of cerebral arteries |
| G64z.00 | 3149 | Cerebral infarction |
| G64z.11 | 15252 | Brainstem infarction |
| G64z.12 | 5602 | Cerebellar infarction |
| G64z000 | 25615 | Brainstem infarction |
| G64z100 | 47642 | Wallenberg syndrome |
| G64z111 | 5185 | Lateral medullary syndrome |
| G64z200 | 9985 | Left sided cerebral infarction |
| G64z300 | 10504 | Right sided cerebral infarction |
| G64z400 | 26424 | Infarction of basal ganglia |
| G665.00 | 33499 | Pure motor lacunar syndrome |
| G666.00 | 51767 | Pure sensory lacunar syndrome |
| G676000 | 39344 | Cerebral infarction due to cerebral venous thrombosis, nonpyogenic |
| G6W..00 | 40758 | Cerebral infarction due to unspecified occlusion/stenosis of precerebral arteries |
| Gyu6300 | 91627 | Cerebral infarction due to unspecified occlusion or stenosis of cerebral arteries |
| Gyu6400 | 53745 | Other cerebral infarction |
| Gyu6500 | 90572 | Occlusion and stenosis of other precerebral arteries |
| Gyu6600 | 92036 | Occlusion and stenosis of other cerebral arteries |
| Gyu6G00 | 94482 | Cerebral infarction due to unspecified occlusion/stenosis of precerebral arteries |
| 14A7.00 | 34135 | CVA/stroke |
| 14A7.11 | 6305 | CVA |
| 14A7.12 | 5871 | Stroke |
| 14AK.00 | 66873 | Stroke in last year |
| ZV12511 | 19348 | Personal history of stroke |
| ZV12512 | 7138 | Personal history of cerebrovascular accident (CVA) |
| Fyu5500 | 63746 | Other transient cerebral ischemic attacks and related syndromes |
| G65..00 | 504 | Transient cerebral ischemia |
| G65..11 | 3132 | Drop attack |
| G65..12 | 1433 | Transient ischemic attack |
| G65..13 | 2417 | Vertebro-basilar insufficiency |
| G650.00 | 23942 | Basilar artery syndrome |
| G650.11 | 5268 | Insufficiency - basilar artery |
| G651.00 | 33377 | Vertebral artery syndrome |
| G651000 | 21118 | Vertebro-basilar artery syndrome |
| G652.00 | 23465 | Subclavian steal syndrome |
| G653.00 | 44765 | Carotid artery syndrome hemispheric |
| G654.00 | 50594 | Multiple and bilateral precerebral artery syndromes |
| G655.00 | 6489 | Transient global amnesia |
| G656.00 | 10794 | Vertebrobasilar insufficiency |
| G657.00 | 105738 | Carotid territory transient ischemic attack |
| G65y.00 | 19354 | Other transient cerebral ischemia |
| G65z.00 | 1895 | Transient cerebral ischemia |
| G65z000 | 55247 | Impending cerebral ischemia |
| G65z100 | 16507 | Intermittent cerebral ischemia |
| G65zz00 | 15788 | Transient cerebral ischemia |
| Zv12D00 | 101251 | Personal history of transient ischemic attack |
| 14AB.00 | 13567 | TIA |
| **EUROASPIRE Coronary Event** | | |
| G30..00 | 241 | Acute myocardial infarction |
| G300.00 | 12139 | Acute anterolateral infarction |
| G30..11 | 13566 | Attack – heart |
| G30..12 | 2491 | Coronary thrombosis |
| G30..14 | 1204 | Heart attack |
| G30..15 | 1677 | MI - acute myocardial infarction |
| G30..16 | 13571 | Thrombosis - coronary |
| G30..17 | 17689 | Silent myocardial infarction |
| G301.00 | 5387 | Other specified anterior myocardial infarction |
| G301000 | 40429 | Acute anteroapical infarction |
| G301100 | 17872 | Acute anteroseptal infarction |
| G301z00 | 14897 | Anterior myocardial infarction |
| G302.00 | 8935 | Acute inferolateral infarction |
| G303.00 | 29643 | Acute inferoposterior infarction |
| G304.00 | 23892 | Posterior myocardial infarction |
| G305.00 | 14898 | Lateral myocardial infarction |
| G306.00 | 63467 | True posterior myocardial infarction |
| G307.00 | 3704 | Acute subendocardial infarction |
| G307000 | 9507 | Acute non-Q wave infarction |
| G307100 | 10562 | Acute non-ST segment elevation myocardial infarction |
| G308.00 | 1678 | Inferior myocardial infarction |
| G309.00 | 30330 | Acute Q-wave infarct |
| G30B.00 | 32854 | Acute posterolateral myocardial infarction |
| G30X.00 | 29758 | Acute transmural myocardial infarction of unspecified site |
| G30X000 | 12229 | Acute ST segment elevation myocardial infarction |
| G30y.00 | 34803 | Other acute myocardial infarction |
| G30y000 | 28736 | Acute atrial infarction |
| G30y100 | 62626 | Acute papillary muscle infarction |
| G30y200 | 41221 | Acute septal infarction |
| G30yz00 | 46017 | Other acute myocardial infarction |
| G30z.00 | 14658 | Acute myocardial infarction |
| G30A.00 | 17133 | Mural thrombosis |
| G30..13 | 30421 | Cardiac rupture following myocardial infarction (MI) |
| 323..00 | 7783 | ECG: myocardial infarction |
| 3233.00 | 26975 | ECG: antero-septal infarction |
| 3234.00 | 26972 | ECG: posterior/inferior infarction |
| 3235.00 | 55401 | ECG: subendocardial infarction |
| 3236.00 | 52705 | ECG: lateral infarction |
| 323Z.00 | 59032 | ECG: myocardial infarct |
| G311500 | 11983 | Acute coronary syndrome |
| G38..00 | 32272 | Postoperative myocardial infarction |
| G380.00 | 46112 | Postoperative transmural myocardial infarction anterior wall |
| G381.00 | 46276 | Postoperative transmural myocardial infarction inferior wall |
| G383.00 | 106812 | Postoperative transmural myocardial infarction unspecified site |
| G384.00 | 41835 | Postoperative subendocardial myocardial infarction |
| G38z.00 | 68748 | Postoperative myocardial infarction, unspecified |
| 14A3.00 | 35674 | Myocardial infarction <60 |
| 14A4.00 | 40399 | Myocardial infarction >60 |
| 14AH.00 | 50372 | Myocardial infarction in last year |
| 3232.00 | 39904 | ECG: old myocardial infarction |
| G32..00 | 4017 | Old myocardial infarction |
| G32..11 | 16408 | Healed myocardial infarction |
| G32..12 | 17464 | Personal history of myocardial infarction |
| G3...00 | 240 | Ischemic heart disease |
| G3...11 | 24783 | Arteriosclerotic heart disease |
| G3...12 | 20416 | Atherosclerotic heart disease |
| G3...13 | 1792 | IHD - Ischemic heart disease |
| G31..00 | 27951 | Other acute and subacute ischemic heart disease |
| G311.00 | 36523 | Preinfarction syndrome |
| G311.11 | 4656 | Crescendo angina |
| G311.12 | 39655 | Impending infarction |
| G311.13 | 1431 | Unstable angina |
| G311.14 | 19655 | Angina at rest |
| G311000 | 61072 | Myocardial infarction aborted |
| G311011 | 55137 | MI - myocardial infarction aborted |
| G311100 | 7347 | Unstable angina |
| G311200 | 17307 | Angina at rest |
| G311300 | 34328 | Refractory angina |
| G311400 | 18118 | Worsening angina |
| G311z00 | 54251 | Preinfarction syndrome |
| G312.00 | 39449 | Coronary thrombosis not resulting in myocardial infarction |
| G31y.00 | 9413 | Other acute and subacute ischemic heart disease |
| G31y000 | 9276 | Acute coronary insufficiency |
| G31y100 | 8357 | Microinfarction of heart |
| G31y200 | 39693 | Subendocardial ischemia |
| G31y300 | 21844 | Transient myocardial ischemia |
| G31yz00 | 27977 | Other acute and subacute ischemic heart disease |
| G33..00 | 1430 | Angina pectoris |
| G330.00 | 20095 | Angina decubitus |
| G330000 | 18125 | Nocturnal angina |
| G330z00 | 29902 | Angina decubitus |
| G33z.00 | 25842 | Angina pectoris |
| G33z000 | 66388 | Status anginosus |
| G33z100 | 54535 | Stenocardia |
| G33z200 | 7696 | Syncope anginosa |
| G33z300 | 1414 | Angina on effort |
| G33z400 | 32450 | Ischemic chest pain |
| G33z500 | 9555 | Post infarct angina |
| G33z600 | 26863 | New onset angina |
| G33z700 | 12804 | Stable angina |
| G33zz00 | 28554 | Angina pectoris |
| G34..00 | 28138 | Other chronic ischemic heart disease |
| G340.00 | 5413 | Coronary atherosclerosis |
| G340.11 | 1655 | Triple vessel disease of the heart |
| G340.12 | 1344 | Coronary artery disease |
| G340000 | 3999 | Single coronary vessel disease |
| G340100 | 5254 | Double coronary vessel disease |
| G342.00 | 36609 | Atherosclerotic cardiovascular disease |
| G343.00 | 7320 | Ischemic cardiomyopathy |
| G344.00 | 29421 | Silent myocardial ischemia |
| G34y.00 | 34633 | Other specified chronic ischemic heart disease |
| G34y000 | 24540 | Chronic coronary insufficiency |
| G34y100 | 23078 | Chronic myocardial ischemia |
| G34yz00 | 35713 | Other specified chronic ischemic heart disease |
| G34z.00 | 15754 | Other chronic ischemic heart disease |
| G34z000 | 18889 | Asymptomatic coronary heart disease |
| G35..00 | 18842 | Subsequent myocardial infarction |
| G350.00 | 45809 | Subsequent myocardial infarction of anterior wall |
| G351.00 | 38609 | Subsequent myocardial infarction of inferior wall |
| G353.00 | 72562 | Subsequent myocardial infarction of other sites |
| G35X.00 | 46166 | Subsequent myocardial infarction of unspecified site |
| G39..00 | 105479 | Coronary microvascular disease |
| G3y..00 | 22383 | Other specified ischemic heart disease |
| G3z..00 | 1676 | Ischemic heart disease |
| Gyu3.00 | 52517 | Ischemic heart diseases |
| Gyu3000 | 39546 | Other forms of angina pectoris |
| Gyu3200 | 68401 | Other forms of acute ischemic heart disease |
| Gyu3300 | 47637 | Other forms of chronic ischemic heart disease |
| Gyu3400 | 96838 | Acute transmural myocardial infarction of unspecified site |
| Gyu3600 | 99991 | Subsequent myocardial infarction of unspecified site |
| 792..11 | 737 | Coronary artery bypass graft operations |
| 7920.00 | 18249 | Saphenous vein graft replacement of coronary artery |
| 7920.11 | 8312 | Saphenous vein graft bypass of coronary artery |
| 7920000 | 8679 | Saphenous vein graft replacement of one coronary artery |
| 7920100 | 7634 | Saphenous vein graft replacement of two coronary arteries |
| 7920200 | 7442 | Saphenous vein graft replacement of three coronary arteries |
| 7920300 | 11610 | Saphenous vein graft replacement of four+ coronary arteries |
| 7920y00 | 7137 | Saphenous vein graft replacement of coronary artery |
| 7920z00 | 51515 | Saphenous vein graft replacement coronary artery |
| 7921.00 | 9414 | Other autograft replacement of coronary artery |
| 7921.11 | 7134 | Other autograft bypass of coronary artery |
| 7921000 | 44561 | Autograft replacement of one coronary artery |
| 7921100 | 19413 | Autograft replacement of two coronary arteries |
| 7921200 | 10209 | Autograft replacement of three coronary arteries |
| 7921300 | 42708 | Autograft replacement of four of more coronary arteries |
| 7921y00 | 61310 | Other autograft replacement of coronary artery |
| 7921z00 | 7609 | Other autograft replacement of coronary artery |
| 7922.00 | 31556 | Allograft replacement of coronary artery |
| 7922.11 | 32651 | Allograft bypass of coronary artery |
| 7922000 | 70111 | Allograft replacement of one coronary artery |
| 7922100 | 57241 | Allograft replacement of two coronary arteries |
| 7922200 | 45886 | Allograft replacement of three coronary arteries |
| 7922300 | 45370 | Allograft replacement of four or more coronary arteries |
| 7922y00 | 59423 | Other specified allograft replacement of coronary artery |
| 7922z00 | 48767 | Allograft replacement of coronary artery |
| 7923.00 | 19402 | Prosthetic replacement of coronary artery |
| 7923.11 | 36011 | Prosthetic bypass of coronary artery |
| 7923000 | 92419 | Prosthetic replacement of one coronary artery |
| 7923100 | 66664 | Prosthetic replacement of two coronary arteries |
| 7923200 | 66236 | Prosthetic replacement of three coronary arteries |
| 7923300 | 67761 | Prosthetic replacement of four or more coronary arteries |
| 7923z00 | 19193 | Prosthetic replacement of coronary artery |
| 7924.00 | 33461 | Revision of bypass for coronary artery |
| 7924000 | 52938 | Revision of bypass for one coronary artery |
| 7924100 | 67554 | Revision of bypass for two coronary arteries |
| 7924200 | 31540 | Revision of bypass for three coronary arteries |
| 7924300 | 101569 | Revision of bypass for four or more coronary arteries |
| 7924500 | 63153 | Revision of implantation of thoracic artery into heart |
| 7924y00 | 97953 | Other specified revision of bypass for coronary artery |
| 7924z00 | 57634 | Revision of bypass for coronary artery |
| 7925.00 | 37682 | Connection of mammary artery to coronary artery |
| 7925.11 | 28837 | Creation of bypass from mammary artery to coronary artery |
| 7925000 | 33718 | Double anastomosis of mammary arteries to coronary arteries |
| 7925011 | 48822 | LIMA sequential anastomosis |
| 7925012 | 92233 | RIMA sequential anastomosis |
| 7925100 | 31519 | Double implant of mammary arteries into coronary arteries |
| 7925200 | 44723 | Single anastomosis mammary art to left ant descend coronary art |
| 7925300 | 51507 | Single anastomosis of mammary artery to coronary artery |
| 7925311 | 22647 | LIMA single anastomosis |
| 7925312 | 68123 | RIMA single anastomosis |
| 7925400 | 68139 | Single implantation of mammary artery into coronary artery |
| 7925y00 | 37719 | Connection of mammary artery to coronary artery |
| 7925z00 | 56990 | Connection of mammary artery to coronary artery |
| 7926.00 | 96804 | Connection of other thoracic artery to coronary artery |
| 7926000 | 62608 | Double anastomosis thoracic arteries to coronary arteries |
| 7926200 | 67591 | Single anastomosis of thoracic artery to coronary artery |
| 7926300 | 60753 | Single implantation thoracic artery into coronary artery |
| 7926z00 | 72780 | Connection of other thoracic artery to coronary artery |
| 792C.00 | 55598 | Other replacement of coronary artery |
| 792C000 | 55092 | Replacement of coronary arteries using multiple methods |
| 792Cy00 | 93828 | Other specified replacement of coronary artery |
| 792Cz00 | 70755 | Replacement of coronary artery |
| 792D.00 | 34963 | Other bypass of coronary artery |
| 792Dy00 | 3159 | Other specified other bypass of coronary artery |
| 792Dz00 | 33471 | Other bypass of coronary artery |
| 792y.00 | 31571 | Other specified operations on coronary artery |
| 790H300 | 12743 | Revascularization of wall of heart |
| 7927500 | 5744 | Open angioplasty of coronary artery |
| 7928.00 | 2901 | Transluminal balloon angioplasty of coronary artery |
| 7928.11 | 5703 | Percutaneous balloon coronary angioplasty |
| 7928000 | 18670 | Percutaneous transluminal balloon angioplasty one coronary artery |
| 7928100 | 33735 | Percutaneous transluminal balloon angioplasty multiple coronary arteries |
| 7928200 | 42462 | Percutaneous transluminal balloon angioplasty bypass graft coronary artery |
| 7928300 | 86071 | Percutaneous transluminal cutting balloon angioplasty coronary artery |
| 7928y00 | 41547 | Transluminal balloon angioplasty of coronary artery |
| 7928z00 | 732 | Transluminal balloon angioplasty of coronary artery |
| 7929.00 | 24888 | Other therapeutic transluminal operations on coronary artery |
| 7929000 | 22828 | Percutaneous transluminal laser coronary angioplasty |
| 7929100 | 33650 | Percutaneous transluminal coronary thrombolysis with streptokinase |
| 7929111 | 40996 | Percutaneous transluminal coronary thrombolytic therapy- streptokinase |
| 7929200 | 66583 | Percutaneous transluminal injection therapy substitute to coronary artery |
| 7929300 | 19046 | Rotary blade coronary angioplasty |
| 7929400 | 8942 | Insertion of coronary artery stent |
| 7929500 | 42304 | Insertion of drug-eluting coronary artery stent |
| 7929600 | 93618 | Percutaneous transluminal atherectomy of coronary artery |
| 7929y00 | 6182 | Other therapeutic transluminal operation on coronary artery |
| 7929z00 | 31679 | Other therapeutic transluminal operation on coronary artery |
| 793G.00 | 43939 | Percutaneous transluminal balloon angioplasty stenting coronary artery |
| 793G000 | 60067 | Percutaneous transluminal ball angioplasty insert 1-2 drug stents coronary artery |
| 793G100 | 87849 | Percutaneous transluminal ball angioplasty ins 3 or more drug stents coronary artery |
| 793G200 | 85947 | Percutaneous transluminal balloon angioplasty insert 1-2 stents coronary artery |
| 793G300 | 92927 | Percutaneous coronary balloon angioplasty 3 more stents coronary artery |
| 793Gy00 | 96537 | Percutaneous transluminal balloon angioplasty stenting coronary artery |
| 793Gz00 | 61208 | Percutaneous transluminal balloon angioplasty stenting coronary artery |
| ZV45L00 | 6980 | Status following coronary angioplasty |

**Supplemental Methods Table 5. Description of study variables, associated data sources and method of assessment.**

| **Variables**  **category** | **Description** |
| --- | --- |
| Practice characteristics | Primary Care Practices were defined by region and had to have research-active and UTS (Up-to-Standard) status at the time of analysis (UTS is an internal CPRD quality indicator derived from an algorithm examining practice death recording and gaps in data). |
| Patient demographics | All Patient level Data are fully anonymized. Variables used in analyses included: Age, Gender and Ethnicity. |
| Clinical characteristics | Read version (v) 2 codes and associated descriptions of medical codes, referenced in the data files as ‘Medcode’, were used to define all diagnoses and comorbidities of interest. |
| Laboratory Test information | Lab test results were derived using the combination of Entity Type (Enttype) and Medcode (with description) corresponding to the specific lab test or examination performed, the date it was performed, and the test result(s). For example, Serum Cholesterol has the Entity type ‘163’ with associated Medcodes, such as: ‘12’ (Serum Cholesterol); ‘13733’ (Serum Total Cholesterol Level); ‘18147’ (Total Cholesterol measurement). The test result also contains details such as the units of each specific lab test (e.g. mmol/l or mg/dL for Serum Cholesterol). |
| Medication use | Gemscript product codes (brand and generic name) and associated descriptions of medication/product codes (referenced in the data files as ‘prodcode’) were used to define medication use. |
| Data linkage | Supplemental information was used for patients in English Primary Care Practices using Hospital Episode Statistics (HES) Admitted Care and Outpatient data, and Office for National Statistics (ONS) Mortality linked data. HES and ONS databases use ICD-10 codes. |

**Supplemental Methods Table 6. Causes of death included under ‘mortality attributable to circulatory causes’.**

**Diseases of the Circulatory System (ICD-10: I00-I99)**

| **ICD-10** | | **Description** |
| --- | --- | --- |
| **I00** | Rheumatic fever without mention of heart involvement | |
| **I01** | Rheumatic fever with heart involvement | |
| **I010** | Acute rheumatic pericarditis | |
| **I011** | Acute rheumatic endocarditis | |
| **I012** | Acute rheumatic myocarditis | |
| **I018** | Other acute rheumatic heart disease | |
| **I019** | Acute rheumatic heart disease, unspecified | |
| **I02** | Rheumatic chorea | |
| **I020** | Rheumatic chorea with heart involvement | |
| **I029** | Rheumatic chorea without heart involvement | |
| **I05** | Rheumatic mitral valve diseases | |
| **I050** | Mitral stenosis | |
| **I051** | Rheumatic mitral insufficiency | |
| **I052** | Mitral stenosis with insufficiency | |
| **I058** | Other mitral valve diseases | |
| **I059** | Mitral valve disease, unspecified | |
| **I06** | Rheumatic aortic valve diseases | |
| **I060** | Rheumatic aortic stenosis | |
| **I061** | Rheumatic aortic insufficiency | |
| **I062** | Rheumatic aortic stenosis with insufficiency | |
| **I068** | Other rheumatic aortic valve diseases | |
| **I069** | Rheumatic aortic valve disease, unspecified | |
| **I07** | Rheumatic tricuspid valve diseases | |
| **I070** | Tricuspid stenosis | |
| **I071** | Tricuspid insufficiency | |
| **I072** | Tricuspid stenosis with insufficiency | |
| **I078** | Other tricuspid valve diseases | |
| **I079** | Tricuspid valve disease, unspecified | |
| **I08** | Multiple valve diseases | |
| **I080** | Disorders of both mitral and aortic valves | |
| **I081** | Disorders of both mitral and tricuspid valves | |
| **I082** | Disorders of both aortic and tricuspid valves | |
| **I083** | Combined disorders of mitral, aortic and tricuspid valves | |
| **I088** | Other multiple valve diseases | |
| **I089** | Multiple valve disease, unspecified | |
| **I09** | Other rheumatic heart diseases | |
| **I090** | Rheumatic myocarditis | |
| **I091** | Rheumatic diseases of endocardium, valve unspecified | |
| **I092** | Chronic rheumatic pericarditis | |
| **I098** | Other specified rheumatic heart diseases | |
| **I099** | Rheumatic heart disease, unspecified | |
| **I10** | Essential (primary) hypertension | |
| **I11** | Hypertensive heart disease | |
| **I110** | Hypertensive heart disease with (congestive) heart failure | |
| **I119** | Hypertensive heart disease without (congestive) heart failure | |
| **I12** | Hypertensive renal disease | |
| **I120** | Hypertensive renal disease with renal failure | |
| **I129** | Hypertensive renal disease without renal failure | |
| **I13** | Hypertensive heart and renal disease | |
| **I130** | Hypertensive heart and renal disease with (congestive) heart failure | |
| **I131** | Hypertensive heart and renal disease with renal failure | |
| **I132** | Hypertensive heart and renal disease with both (congestive) heart failure and renal failure | |
| **I139** | Hypertensive heart and renal disease, unspecified | |
| **I15** | Secondary hypertension | |
| **I150** | Renovascular hypertension | |
| **I151** | Hypertension secondary to other renal disorders | |
| **I152** | Hypertension secondary to endocrine disorders | |
| **I158** | Other secondary hypertension | |
| **I159** | Secondary hypertension, unspecified | |
| **I20** | Angina pectoris | |
| **I200** | Unstable angina | |
| **I201** | Angina pectoris with documented spasm | |
| **I208** | Other forms of angina pectoris | |
| **I209** | Angina pectoris, unspecified | |
| **I21** | Acute myocardial infarction | |
| **I210** | Acute transmural myocardial infarction of anterior wall | |
| **I211** | Acute transmural myocardial infarction of inferior wall | |
| **I212** | Acute transmural myocardial infarction of other sites | |
| **I213** | Acute transmural myocardial infarction of unspecified site | |
| **I214** | Acute subendocardial myocardial infarction | |
| **I219** | Acute myocardial infarction, unspecified | |
| **I22** | Subsequent myocardial infarction | |
| **I220** | Subsequent myocardial infarction of anterior wall | |
| **I221** | Subsequent myocardial infarction of inferior wall | |
| **I228** | Subsequent myocardial infarction of other sites | |
| **I229** | Subsequent myocardial infarction of unspecified site | |
| **I23** | Certain current complications following acute myocardial infarction | |
| **I230** | Haemopericardium as current complication following acute MI | |
| **I231** | Atrial septal defect as current complication following acute MI | |
| **I232** | Ventricular septal defect as current complication following acute MI | |
| **I233** | Rupture of cardiac wall without haemopericardium as current complication following acute MI | |
| **I234** | Rupture of chordae tendineae as current complication following acute MI | |
| **I235** | Rupture of papillary muscle as current complication following acute MI | |
| **I236** | Thrombosis of atrium, auricular appendage, and ventricle as current complications following acute MI | |
| **I238** | Other current complications following acute MI | |
| **I24** | Other acute ischaemic heart diseases | |
| **I240** | Coronary thrombosis not resulting in myocardial infarction | |
| **I241** | Dressler syndrome | |
| **I248** | Other forms of acute ischaemic heart disease | |
| **I249** | Acute ischaemic heart disease, unspecified | |
| **I25** | Chronic ischaemic heart disease | |
| **I250** | Atherosclerotic cardiovascular disease, so described | |
| **I251** | Atherosclerotic heart disease | |
| **I252** | Old myocardial infarction | |
| **I253** | Aneurysm of heart | |
| **I254** | Coronary artery aneurysm and dissection | |
| **I255** | Ischaemic cardiomyopathy | |
| **I256** | Silent myocardial ischaemia | |
| **I258** | Other forms of chronic ischaemic heart disease | |
| **I259** | Chronic ischaemic heart disease, unspecified | |
| **I26** | Pulmonary embolism | |
| **I260** | Pulmonary embolism with mention of acute cor pulmonale | |
| **I269** | Pulmonary embolism without mention of acute cor pulmonale | |
| **I27** | Other pulmonary heart diseases | |
| **I270** | Primary pulmonary hypertension | |
| **I271** | Kyphoscoliotic heart disease | |
| **I272** | Other secondary pulmonary hypertension | |
| **I278** | Other specified pulmonary heart diseases | |
| **I279** | Pulmonary heart disease, unspecified | |
| **I28** | Other diseases of pulmonary vessels | |
| **I280** | Arteriovenous fistula of pulmonary vessels | |
| **I281** | Aneurysm of pulmonary artery | |
| **I288** | Other specified diseases of pulmonary vessels | |
| **I289** | Disease of pulmonary vessels, unspecified | |
| **I30** | Acute pericarditis | |
| **I300** | Acute nonspecific idiopathic pericarditis | |
| **I301** | Infective pericarditis | |
| **I308** | Other forms of acute pericarditis | |
| **I309** | Acute pericarditis, unspecified | |
| **I31** | Other diseases of pericardium | |
| **I310** | Chronic adhesive pericarditis | |
| **I311** | Chronic constrictive pericarditis | |
| **I312** | Haemopericardium, not elsewhere classified | |
| **I313** | Pericardial effusion (noninflammatory) | |
| **I318** | Other specified diseases of pericardium | |
| **I319** | Disease of pericardium, unspecified | |
| **I32** | Pericarditis in diseases classified elsewhere | |
| **I320** | Pericarditis in bacterial diseases classified elsewhere | |
| **I321** | Pericarditis in other infectious and parasitic diseases classified elsewhere | |
| **I328** | Pericarditis in other diseases classified elsewhere | |
| **I33** | Acute and subacute endocarditis | |
| **I330** | Acute and subacute infective endocarditis | |
| **I339** | Acute endocarditis, unspecified | |
| **I34** | Nonrheumatic mitral valve disorders | |
| **I340** | Mitral (valve) insufficiency | |
| **I341** | Mitral (valve) prolapse | |
| **I342** | Nonrheumatic mitral (valve) stenosis | |
| **I348** | Other nonrheumatic mitral valve disorders | |
| **I349** | Nonrheumatic mitral valve disorder, unspecified | |
| **I35** | Nonrheumatic aortic valve disorders | |
| **I350** | Aortic (valve) stenosis | |
| **I351** | Aortic (valve) insufficiency | |
| **I352** | Aortic (valve) stenosis with insufficiency | |
| **I358** | Other aortic valve disorders | |
| **I359** | Aortic valve disorder, unspecified | |
| **I36** | Nonrheumatic tricuspid valve disorders | |
| **I360** | Nonrheumatic tricuspid (valve) stenosis | |
| **I361** | Nonrheumatic tricuspid (valve) insufficiency | |
| **I362** | Nonrheumatic tricuspid (valve) stenosis with insufficiency | |
| **I368** | Other nonrheumatic tricuspid valve disorders | |
| **I369** | Nonrheumatic tricuspid valve disorder, unspecified | |
| **I37** | Pulmonary valve disorders | |
| **I370** | Pulmonary valve stenosis | |
| **I371** | Pulmonary valve insufficiency | |
| **I372** | Pulmonary valve stenosis with insufficiency | |
| **I378** | Other pulmonary valve disorders | |
| **I379** | Pulmonary valve disorder, unspecified | |
| **I38** | Endocarditis, valve unspecified | |
| **I39** | Endocarditis and heart valve disorders in diseases classified elsewhere | |
| **I390** | Mitral valve disorders in diseases classified elsewhere | |
| **I391** | Aortic valve disorders in diseases classified elsewhere | |
| **I392** | Tricuspid valve disorders in diseases classified elsewhere | |
| **I393** | Pulmonary valve disorders in diseases classified elsewhere | |
| **I394** | Multiple valve disorders in diseases classified elsewhere | |
| **I398** | Endocarditis, valve unspecified, in diseases classified elsewhere | |
| **I40** | Acute myocarditis | |
| **I400** | Infective myocarditis | |
| **I401** | Isolated myocarditis | |
| **I408** | Other acute myocarditis | |
| **I409** | Acute myocarditis, unspecified | |
| **I41** | Myocarditis in diseases classified elsewhere | |
| **I410** | Myocarditis in bacterial diseases classified elsewhere | |
| **I411** | Myocarditis in viral diseases classified elsewhere | |
| **I412** | Myocarditis in other infectious and parasitic diseases classified elsewhere | |
| **I418** | Myocarditis in other diseases classified elsewhere | |
| **I42** | Cardiomyopathy | |
| **I420** | Dilated cardiomyopathy | |
| **I421** | Obstructive hypertrophic cardiomyopathy | |
| **I422** | Other hypertrophic cardiomyopathy | |
| **I423** | Endomyocardial (eosinophilic) disease | |
| **I424** | Endocardial fibroelastosis | |
| **I425** | Other restrictive cardiomyopathy | |
| **I426** | Alcoholic cardiomyopathy | |
| **I427** | Cardiomyopathy due to drugs and other external agents | |
| **I428** | Other cardiomyopathies | |
| **I429** | Cardiomyopathy, unspecified | |
| **I43** | Cardiomyopathy in diseases classified elsewhere | |
| **I430** | Cardiomyopathy in infectious and parasitic diseases classified elsewhere | |
| **I431** | Cardiomyopathy in metabolic diseases | |
| **I432** | Cardiomyopathy in nutritional diseases | |
| **I438** | Cardiomyopathy in other diseases classified elsewhere | |
| **I44** | Atrioventricular and left bundle-branch block | |
| **I440** | Atrioventricular block, first degree | |
| **I441** | Atrioventricular block, second degree | |
| **I442** | Atrioventricular block, complete | |
| **I443** | Other and unspecified atrioventricular block | |
| **I444** | Left anterior fascicular block | |
| **I445** | Left posterior fascicular block | |
| **I446** | Other and unspecified fascicular block | |
| **I447** | Left bundle-branch block, unspecified | |
| **I45** | Other conduction disorders | |
| **I450** | Right fascicular block | |
| **I451** | Other and unspecified right bundle-branch block | |
| **I452** | Bifascicular block | |
| **I453** | Trifascicular block | |
| **I454** | Nonspecific intraventricular block | |
| **I455** | Other specified heart block | |
| **I456** | Pre-excitation syndrome | |
| **I458** | Other specified conduction disorders | |
| **I459** | Conduction disorder, unspecified | |
| **I46** | Cardiac arrest | |
| **I460** | Cardiac arrest with successful resuscitation | |
| **I461** | Sudden cardiac death, so described | |
| **I469** | Cardiac arrest, unspecified | |
| **I47** | Paroxysmal tachycardia | |
| **I470** | Re-entry ventricular arrhythmia | |
| **I471** | Supraventricular tachycardia | |
| **I472** | Ventricular tachycardia | |
| **I479** | Paroxysmal tachycardia, unspecified | |
| **I48** | Atrial fibrillation and flutter | |
| **I480** | Paroxysmal atrial fibrillation | |
| **I481** | Persistent atrial fibrillation | |
| **I482** | Chronic atrial fibrillation | |
| **I483** | Typical atrial flutter | |
| **I484** | Atypical atrial flutter | |
| **I489** | Atrial fibrillation and atrial flutter, unspecified | |
| **I49** | Other cardiac arrhythmias | |
| **I490** | Ventricular fibrillation and flutter | |
| **I491** | Atrial premature depolarization | |
| **I492** | Junctional premature depolarization | |
| **I493** | Ventricular premature depolarization | |
| **I494** | Other and unspecified premature depolarization | |
| **I495** | Sick sinus syndrome | |
| **I498** | Other specified cardiac arrhythmias | |
| **I499** | Cardiac arrhythmia, unspecified | |
| **I50** | Heart failure | |
| **I500** | Congestive heart failure | |
| **I501** | Left ventricular failure | |
| **I509** | Heart failure, unspecified | |
| **I51** | Complications and ill-defined descriptions of heart disease | |
| **I510** | Cardiac septal defect, acquired | |
| **I511** | Rupture of chordae tendineae, not elsewhere classified | |
| **I512** | Rupture of papillary muscle, not elsewhere classified | |
| **I513** | Intracardiac thrombosis, not elsewhere classified | |
| **I514** | Myocarditis, unspecified | |
| **I515** | Myocardial degeneration | |
| **I516** | Cardiovascular disease, unspecified | |
| **I517** | Cardiomegaly | |
| **I518** | Other ill-defined heart diseases | |
| **I519** | Heart disease, unspecified | |
| **I52** | Other heart disorders in diseases classified elsewhere | |
| **I520** | Other heart disorders in bacterial diseases classified elsewhere | |
| **I521** | Other heart disorders in other infectious and parasitic diseases classified elsewhere | |
| **I528** | Other heart disorders in other diseases classified elsewhere | |
| **I60** | Subarachnoid haemorrhage | |
| **I600** | Subarachnoid haemorrhage from carotid siphon and bifurcation | |
| **I601** | Subarachnoid haemorrhage from middle cerebral artery | |
| **I602** | Subarachnoid haemorrhage from anterior communicating artery | |
| **I603** | Subarachnoid haemorrhage from posterior communicating artery | |
| **I604** | Subarachnoid haemorrhage from basilar artery | |
| **I605** | Subarachnoid haemorrhage from vertebral artery | |
| **I606** | Subarachnoid haemorrhage from other intracranial arteries | |
| **I607** | Subarachnoid haemorrhage from intracranial artery, unspecified | |
| **I608** | Other subarachnoid haemorrhage | |
| **I609** | Subarachnoid haemorrhage, unspecified | |
| **I61** | Intracerebral haemorrhage | |
| **I610** | Intracerebral haemorrhage in hemisphere, subcortical | |
| **I611** | Intracerebral haemorrhage in hemisphere, cortical | |
| **I612** | Intracerebral haemorrhage in hemisphere, unspecified | |
| **I613** | Intracerebral haemorrhage in brain stem | |
| **I614** | Intracerebral haemorrhage in cerebellum | |
| **I615** | Intracerebral haemorrhage, intraventricular | |
| **I616** | Intracerebral haemorrhage, multiple localized | |
| **I618** | Other intracerebral haemorrhage | |
| **I619** | Intracerebral haemorrhage, unspecified | |
| **I62** | Other nontraumatic intracranial haemorrhage | |
| **I620** | Nontraumatic subdural haemorrhage | |
| **I621** | Nontraumatic extradural haemorrhage | |
| **I629** | Intracranial haemorrhage (nontraumatic), unspecified | |
| **I63** | Cerebral infarction | |
| **I630** | Cerebral infarction due to thrombosis of precerebral arteries | |
| **I631** | Cerebral infarction due to embolism of precerebral arteries | |
| **I632** | Cerebral infarction due to unspecified occlusion or stenosis of precerebral arteries | |
| **I633** | Cerebral infarction due to thrombosis of cerebral arteries | |
| **I634** | Cerebral infarction due to embolism of cerebral arteries | |
| **I635** | Cerebral infarction due to unspecified occlusion or stenosis of cerebral arteries | |
| **I636** | Cerebral infarction due to cerebral venous thrombosis, nonpyogenic | |
| **I638** | Other cerebral infarction | |
| **I639** | Cerebral infarction, unspecified | |
| **I64** | Stroke, not specified as haemorrhage or infarction | |
| **I65** | Occlusion and stenosis of precerebral arteries, not resulting in cerebral infarction | |
| **I650** | Occlusion and stenosis of vertebral artery | |
| **I651** | Occlusion and stenosis of basilar artery | |
| **I652** | Occlusion and stenosis of carotid artery | |
| **I653** | Occlusion and stenosis of multiple and bilateral precerebral arteries | |
| **I658** | Occlusion and stenosis of other precerebral artery | |
| **I659** | Occlusion and stenosis of unspecified precerebral artery | |
| **I66** | Occlusion and stenosis of cerebral arteries, not resulting in cerebral infarction | |
| **I660** | Occlusion and stenosis of middle cerebral artery | |
| **I661** | Occlusion and stenosis of anterior cerebral artery | |
| **I662** | Occlusion and stenosis of posterior cerebral artery | |
| **I663** | Occlusion and stenosis of cerebellar arteries | |
| **I664** | Occlusion and stenosis of multiple and bilateral cerebral arteries | |
| **I668** | Occlusion and stenosis of other cerebral artery | |
| **I669** | Occlusion and stenosis of unspecified cerebral artery | |
| **I67** | Other cerebrovascular diseases | |
| **I670** | Dissection of cerebral arteries, nonruptured | |
| **I671** | Cerebral aneurysm, nonruptured | |
| **I672** | Cerebral atherosclerosis | |
| **I673** | Progressive vascular leukoencephalopathy | |
| **I674** | Hypertensive encephalopathy | |
| **I675** | Moyamoya disease | |
| **I676** | Nonpyogenic thrombosis of intracranial venous system | |
| **I677** | Cerebral arteritis, not elsewhere classified | |
| **I678** | Other specified cerebrovascular diseases | |
| **I679** | Cerebrovascular disease, unspecified | |
| **I68** | Cerebrovascular disorders in diseases classified elsewhere | |
| **I680** | Cerebral amyloid angiopathy E85.- | |
| **I681** | Cerebral arteritis in infectious and parasitic diseases classified elsewhere | |
| **I682** | Cerebral arteritis in other diseases classified elsewhere | |
| **I688** | Other cerebrovascular disorders in diseases classified elsewhere | |
| **I69** | Sequelae of cerebrovascular disease | |
| **I690** | Sequelae of subarachnoid haemorrhage | |
| **I691** | Sequelae of intracerebral haemorrhage | |
| **I692** | Sequelae of other nontraumatic intracranial haemorrhage | |
| **I693** | Sequelae of cerebral infarction | |
| **I694** | Sequelae of stroke, not specified as haemorrhage or infarction | |
| **I698** | Sequelae of other and unspecified cerebrovascular diseases | |
| **I70** | Atherosclerosis | |
| **I700** | Atherosclerosis of aorta | |
| **I701** | Atherosclerosis of renal artery | |
| **I702** | Atherosclerosis of arteries of extremities | |
| **I708** | Atherosclerosis of other arteries | |
| **I709** | Generalized and unspecified atherosclerosis | |
| **I71** | Aortic aneurysm and dissection | |
| **I710** | Dissection of aorta [any part] | |
| **I711** | Thoracic aortic aneurysm, ruptured | |
| **I712** | Thoracic aortic aneurysm, without mention of rupture | |
| **I713** | Abdominal aortic aneurysm, ruptured | |
| **I714** | Abdominal aortic aneurysm, without mention of rupture | |
| **I715** | Thoracoabdominal aortic aneurysm, ruptured | |
| **I716** | Thoracoabdominal aortic aneurysm, without mention of rupture | |
| **I718** | Aortic aneurysm of unspecified site, ruptured | |
| **I719** | Aortic aneurysm of unspecified site, without mention of rupture | |
| **I72** | Other aneurysm and dissection | |
| **I720** | Aneurysm and dissection of carotid artery | |
| **I721** | Aneurysm and dissection of artery of upper extremity | |
| **I722** | Aneurysm and dissection of renal artery | |
| **I723** | Aneurysm and dissection of iliac artery | |
| **I724** | Aneurysm and dissection of artery of lower extremity | |
| **I725** | Aneurysm and dissection of other precerebral arteries | |
| **I726** | Aneurysm and dissection of vertebral artery | |
| **I728** | Aneurysm and dissection of other specified arteries | |
| **I729** | Aneurysm and dissection of unspecified site | |
| **I73** | Other peripheral vascular diseases | |
| **I730** | Raynaud syndrome | |
| **I731** | Thromboangiitis obliterans [Buerger] | |
| **I738** | Other specified peripheral vascular diseases | |
| **I739** | Peripheral vascular disease, unspecified | |
| **I74** | Arterial embolism and thrombosis | |
| **I740** | Embolism and thrombosis of abdominal aorta | |
| **I741** | Embolism and thrombosis of other and unspecified parts of aorta | |
| **I742** | Embolism and thrombosis of arteries of upper extremities | |
| **I743** | Embolism and thrombosis of arteries of lower extremities | |
| **I744** | Embolism and thrombosis of arteries of extremities, unspecified | |
| **I745** | Embolism and thrombosis of iliac artery | |
| **I748** | Embolism and thrombosis of other arteries | |
| **I749** | Embolism and thrombosis of unspecified artery | |
| **I77** | Other disorders of arteries and arterioles | |
| **I770** | Arteriovenous fistula, acquired | |
| **I771** | Stricture of artery | |
| **I772** | Rupture of artery | |
| **I773** | Arterial fibromuscular dysplasia | |
| **I774** | Coeliac artery compression syndrome | |
| **I775** | Necrosis of artery | |
| **I776** | Arteritis, unspecified | |
| **I778** | Other specified disorders of arteries and arterioles | |
| **I779** | Disorder of arteries and arterioles, unspecified | |
| **I78** | Diseases of capillaries | |
| **I780** | Hereditary haemorrhagic telangiectasia | |
| **I781** | Naevus, non-neoplastic | |
| **I788** | Other diseases of capillaries | |
| **I789** | Disease of capillaries, unspecified | |
| **I79** | Disorders of arteries, arterioles and capillaries in diseases classified elsewhere | |
| **I790** | Aneurysm of aorta in diseases classified elsewhere | |
| **I791** | Aortitis in diseases classified elsewhere | |
| **I792** | Peripheral angiopathy in diseases classified elsewhere | |
| **I798** | Other disorders of arteries, arterioles and capillaries in diseases classified elsewhere | |
| **I80** | Phlebitis and thrombophlebitis | |
| **I800** | Phlebitis and thrombophlebitis of superficial vessels of lower extremities | |
| **I801** | Phlebitis and thrombophlebitis of femoral vein | |
| **I802** | Phlebitis and thrombophlebitis of other deep vessels of lower extremities | |
| **I803** | Phlebitis and thrombophlebitis of lower extremities, unspecified | |
| **I808** | Phlebitis and thrombophlebitis of other sites | |
| **I809** | Phlebitis and thrombophlebitis of unspecified site | |
| **I81** | Portal vein thrombosis | |
| **I82** | Other venous embolism and thrombosis | |
| **I820** | Budd-Chiari syndrome | |
| **I821** | Thrombophlebitis migrans | |
| **I822** | Embolism and thrombosis of vena cava | |
| **I823** | Embolism and thrombosis of renal vein | |
| **I828** | Embolism and thrombosis of other specified veins | |
| **I829** | Embolism and thrombosis of unspecified vein | |
| **I83** | Varicose veins of lower extremities | |
| **I830** | Varicose veins of lower extremities with ulcer | |
| **I831** | Varicose veins of lower extremities with inflammation | |
| **I832** | Varicose veins of lower extremities with both ulcer and inflammation | |
| **I839** | Varicose veins of lower extremities without ulcer or inflammation | |
| **I85** | Oesophageal varices | |
| **I850** | Oesophageal varices with bleeding | |
| **I859** | Oesophageal varices without bleeding | |
| **I86** | Varicose veins of other sites | |
| **I860** | Sublingual varices | |
| **I861** | Scrotal varices | |
| **I862** | Pelvic varices | |
| **I863** | Vulval varices | |
| **I864** | Gastric varices | |
| **I868** | Varicose veins of other specified sites | |
| **I87** | Other disorders of veins | |
| **I870** | Postthrombotic syndrome | |
| **I871** | Compression of vein | |
| **I872** | Venous insufficiency (chronic)(peripheral) | |
| **I878** | Other specified disorders of veins | |
| **I879** | Disorder of vein, unspecified | |
| **I88** | Nonspecific lymphadenitis | |
| **I880** | Nonspecific mesenteric lymphadenitis | |
| **I881** | Chronic lymphadenitis, except mesenteric | |
| **I888** | Other nonspecific lymphadenitis | |
| **I889** | Nonspecific lymphadenitis, unspecified | |
| **I89** | Other noninfective disorders of lymphatic vessels and lymph nodes | |
| **I890** | Lymphoedema, not elsewhere classified | |
| **I891** | Lymphangitis | |
| **I898** | Other specified noninfective disorders of lymphatic vessels and lymph nodes | |
| **I899** | Noninfective disorder of lymphatic vessels and lymph nodes, unspecified | |
| **I95** | Hypotension | |
| **I950** | Idiopathic hypotension | |
| **I951** | Orthostatic hypotension | |
| **I952** | Hypotension due to drugs | |
| **I958** | Other hypotension | |
| **I959** | Hypotension, unspecified | |
| **I97** | Postprocedural disorders of circulatory system, not elsewhere classified | |
| **I970** | Postcardiotomy syndrome | |
| **I971** | Other functional disturbances following cardiac surgery | |
| **I972** | Postmastectomy lymphoedema syndrome | |
| **I978** | Other postprocedural disorders of circulatory system, not elsewhere classified | |
| **I979** | Postprocedural disorder of circulatory system, unspecified | |
| **I98** | Other disorders of circulatory system in diseases classified elsewhere | |
| **I980** | Cardiovascular syphilis | |
| **I981** | Cardiovascular disorders in other infectious and parasitic diseases classified elsewhere | |
| **I982** | Oesophageal varices without bleeding in diseases classified elsewhere | |
| **I983** | Oesophageal varices with bleeding in diseases classified elsewhere | |
| **I988** | Other specified disorders of circulatory system in diseases classified elsewhere | |
| **I99** | Other and unspecified disorders of circulatory system | |

## References

1) Toson B, Baker A. 'Life expectancy at birth: methodological options for small populations’, National Statistics Methodological Series No. 33. Office for National Statistics. 2003.

2) Health and Social Care Information Centre (HSCIC), National Health Service (NHS) England. Dataset and Business Rules - Cardiovascular Disease Primary Prevention (CVD-PP) Indicator Set (Retired Indicators) (v29.1). 2014:1–10.

3) Nordestgaard BG, Chapman MJ, Humphries SE, et al. Familial hypercholesterolaemia is underdiagnosed and undertreated in the general population: guidance for clinicians to prevent coronary heart disease: consensus statement of the European Atherosclerosis Society. Eur Heart J 2013;34:3478-90a.

4) Besseling J, Kindt I, Hof M, Kastelein JJ, Hutten BA, Hovingh GK. Severe heterozygous familial hypercholesterolemia and risk for cardiovascular disease: a study of a cohort of 14,000 mutation carriers. Atherosclerosis 2014;233:219-23.

5) De Backer G, Besseling J, Chapman J, et al. Prevalence and management of familial hypercholesterolaemia in coronary patients: An analysis of EUROASPIRE IV, a study of the European Society of Cardiology. Atherosclerosis 2015;241:169-75.

6) Herrett E, Gallagher AM, Bhaskaran K, Forbes H, Mathur R, van Staa T, Smeeth L. Data Resource Profile: Clinical Practice Research Datalink (CPRD). Int J Epidemiol. 2015 Jun;44(3):827-36.
